# Predicting Antarctic Net Snow Accumulation at the Kilometer Scale and Its Impact on Observed Height Changes

**DOI:** 10.1029/2022GL099330

**Published:** 2022-10-17

**Authors:** B. Medley, J. T. M. Lenaerts, M. Dattler, E. Keenan, N. Wever

**Affiliations:** ^1^ Cryospheric Sciences Laboratory NASA Goddard Space Flight Center Greenbelt MD USA; ^2^ Department of Atmospheric and Oceanic Sciences University of Colorado Boulder Boulder CO USA; ^3^ Department of Atmospheric and Oceanic Science University of Maryland College Park College Park MD USA

**Keywords:** Antarctica, surface mass balance, altimetry

## Abstract

Sub‐grid‐scale processes occurring at or near the surface of an ice sheet have a potentially large impact on local and integrated net accumulation of snow via redistribution and sublimation. Given observational complexity, they are either ignored or parameterized over large‐length scales. Here, we train random forest (RF) models to predict variability in net accumulation over the Antarctic Ice Sheet using atmospheric variables and topographic characteristics as predictors at 1 km resolution. Observations of net snow accumulation from both in situ and airborne radar data provide the input observable targets needed to train the RF models. We find that local net accumulation deviates by as much as 172% of the atmospheric model mean. The correlation in space between the predicted net accumulation variability and satellite‐derived surface‐height change indicates that surface processes operate differently through time, driven largely by the seasonal anomalies in snow accumulation.

## Key Points

- We predict spatial deviations in net accumulation through synthesis of topographic and atmospheric characteristics on a 1‐km grid
- Modeled deviations in net accumulation typically range between −41% and 33% and are as large as −172% of the Modern‐Era Retrospective analysis for Research and Applications, Version 2 mean
- The spatiotemporal magnitude and variability in Ice, Cloud, and land Elevation satellite height changes are driven by spatiotemporal anomalies in net accumulation

## 1 Introduction

Large‐scale snowfall events deposit a substantial amount of freshwater over the Antarctic Ice Sheet (AIS), acting in opposition to present‐day sea‐level rise. Approximately 7 mm of global sea‐level equivalent falls annually in the form of snow over the entire ice sheet (Mottram et al., 2021); any short‐to‐long‐term deviations in time and space from this mean will directly impact the temporal evolution mass balance of the AIS and its individual glacial drainage systems (Rignot et al., 2019; Smith et al., 2020). State‐of‐the‐art atmospheric models do not agree, however, on the total magnitude of annual snow accumulation (Mottram et al., 2021), ranging by more 500 Gt yr^−1^, a value which largely overshadows a reconciled AIS total mass balance of −109 Gt yr^−1^ (Shepherd et al., 2018). This lack of constraint yields arguably the largest source of uncertainty in estimates of AIS mass balance and its contribution to global sea level (Rignot et al., 2019; Shepherd et al., 2018; Smith et al., 2020). We aim to constrain the magnitude of net snow accumulation over the AIS at fine spatial resolution within a global atmospheric model using airborne and ground‐based measurements.

While snowfall events over the ice sheet are synoptic, blowing snow processes occurring prior to or after deposition at the surface impart local‐scale variability as snow is redistributed or preferentially sublimated (Lenaerts et al., 2019). At present, global atmospheric models are not capable of accounting for these small‐scale impacts (Gelaro et al., 2017), and only a small handful of Regional climate models (RCMs) simulate these processes albeit at much coarser scales than they actually occur (Amory et al., 2021; Van Wessem et al., 2018). A lack of observed accumulation rates at the scale necessary to measure these local processes challenged development of both physics‐based and empirical models. Recently, ground‐based (Das et al., 2013; Spikes et al., 2004) and airborne (Dattler et al., 2019; Medley et al., 2013) radar observations of the ice sheet's near‐surface internal stratigraphy have revealed the small‐scale variability (SSV) in snow accumulation at fine along‐track resolution and over large swaths of the ice sheet. Here, we built on prior work (Das et al., 2013; Dattler et al., 2019; Scambos et al., 2012; Studinger et al., 2020) investigating SSV in snow accumulation and regions of net scour, including their relationship to local topography and wind characteristics, to predict small‐scale net snow accumulation over the entire ice sheet.

We are only focused on dry snow processes (snowfall, sublimation, erosion, and deposition) that yield net snow accumulation, whereas the surface mass balance (SMB) also accounts for mass loss via runoff. We define net accumulation as the of snow that accumulates at the surface after accounting for all the dry snow processes, and in this work, we allow net accumulation less than zero. Dry snow processes account for almost the entirety of ice‐sheet SMB; runoff is relevant in only small number of areas.

The 1980–2017 mean annual snow accumulation (±1 standard deviation) derived from NASA's Modern‐Era Retrospective analysis for Research and Applications, Version 2 (MERRA‐2; Gelaro et al., 2017) over the AIS totals to 2568 ± 147, with 2037 ± 125 Gt yr^−1^ over grounded ice and 531 ± 34 Gt yr^−1^ over floating ice without accounting for erosion and deposition. This global model is coarsely resolved and does not include physical processes that occur over ice sheets over short length scales, such as blowing snow. RCMs (Agosta et al., 2019; Lenaerts et al., 2012; Van Wessem et al., 2018) have accounted for these processes with varying degrees of complexity; however, while some of the parameterizations hold for transport over smaller length scales (Amory et al., 2021), the model outputs are resolved at the 10s of km scales. For instance, results from a 5 km RCM run over West Antarctica did not show significant improvement in SMB representation against the same RCM run at 27 km (Lenaerts et al., 2018). Another study (Das et al., 2013) used thresholding of wind and topographic regimes to determine regions of net wind scour (i.e., SMB < 0) which yielded an estimated loss of snow mass input due to wind erosion between 11 and 36.5 Gt yr^−1^. The latter study does not provide context for the total impact of snow redistribution because net snow deposition was not considered, providing only one side of the balance equation. Here, we built a static map of net accumulation variability over the grounded and floating portions of the AIS at 1‐km resolution.

## 2 Data

### 2.1 ICESat‐2 Surface Height and Height Change

Launched in 2018, NASA's next generation Ice, Cloud, and land Elevation satellite (ICESat‐2) is a photon‐counting laser altimeter designed to provide precise, repeatable measurements of ice‐surface height change every 91 days, globally to latitudes not exceeding 88° in magnitude (Markus et al., 2017). Here, we use the ICESat‐2 L3A Land Ice Height, Version 2 (ATL06; Smith et al. (2019)) collected during the first three 91‐day cycles (14 October 2018–26 June 2019). Because ICESat‐2 was not pointing at its designed repeat tracks during the first two cycles, data collected during the first ∼180 days provide additional height measurements, which improved spatial coverage. More details regarding building a DEM using ICESat‐2 data are in Text S2 in Supporting Information S1.

To investigate spatial patterns of height change, we also use the ICESat‐2 L3B Slope‐Corrected Land Ice Height Time Series, Version 4 (ATL11; Smith et al. (2021)) spanning cycles 3–11 (29 March 2019–23 June 2021). The ATL11 data set provides along‐track height that is slope‐corrected onto a reference pair track for each cycle beginning with cycle 3 when ICESat‐2 began pointing at its designed reference ground tracks. We eliminate less robust surface heights by using heights that have a quality summary flag set to zero.

### 2.2 Atmospheric

We use several atmospheric variables from NASA's Modern‐Era Retrospective analysis for Research and Applications, Version 2 (MERRA‐2; Gelaro et al., 2017), including hourly 10‐m winds, and snowfall in addition to monthly evaporation, humidity, and surface temperature from 1 January 1980 to 31 December 2019 (GMAO, 2015a, 2015b, 2015c, 2015d). MERRA‐2 data are provided globally at 0.5° latitude by 0.625° longitude resolution.

### 2.3 Snow Accumulation

#### 2.3.1 AntSMB Database

Snow accumulation measurements over the large scales of interest to this work are few. We use a comprehensive collection of Antarctic SMB measurements derived from various sources and methods including ground penetrating radar (GPR), stakes, snow pits and ice cores referred to as the AntSMB data set (Wang et al., 2021). Specifically, we use the multi‐year averaged SMB observations that exceed a 3‐year span, the majority of which are from GPR analysis. Given that the data set contains SMB, there might be some observations where runoff occurs and that are not equivalent to our dry snow net accumulation; however, without a straightforward way to differentiate these sites as well as the relatively small impact of runoff over the AIS, we use all points that meet the time‐span requirement. Thus, we assume net accumulation equals SMB, equivalent to assuming no runoff occurs.

We also derive snow accumulation (Section 4.2.1) from additional snow radar data collected 25 October 2019 (Paden et al., 2014 ) that was released subsequent to the development of the Dattler et al. (2019) data set. We replicate the methodology from Dattler et al. (2019).

#### 2.3.2 Supplemental GPR Measurements

We also use additional GPR data not included in the AntSMB database that were presented by Medley et al. (2014), which cover the Pine Island and Thwaites Glacier catchments. They represent the 1985–2009 mean accumulation rate and are provided at 500‐m along‐track spacing.

## 3 Prediction of Spatial Variability in Snow Accumulation

We built a 1‐km static map of predicted spatial deviations in net accumulation from the background large‐scale MERRA‐2 annual mean using airborne and ground‐based observations of accumulation and a series of topographic and atmospheric predictors. We next outline the various predictors, and then explain the random forest (RF) method implemented for prediction.

### 3.1 Predictors

In all, we used 11 predictors that described the topographic and climatic characteristics as well as their interactions over the AIS. Topographic predictors were based on the DEM described in Text S2 in Supporting Information S1 and include height, slope, aspect, curvature, and a 20‐km high‐pass filter of the surface height (Figures S3–S7 in Supporting Information S1). Because the outer ICESat‐2 beam pairs are separated by ∼6 km, prior to determination of the topographic characteristics, we applied a 6‐km low‐pass filter to the DEM to minimize any tracking artifacts. Climatic predictors were built from MERRA‐2 mean annual variables and include 10‐m wind speed, 10‐m wind direction, air temperature, specific humidity, and total precipitation‐minus‐evaporation (*P* − *E*; Figures S8–S12 in Supporting Information S1). The *P* − *E* is the MERRA‐2 net accumulation. Finally, we use the mean slope in the mean wind direction (Figure S13 in Supporting Information S1), the dot product of the wind and slope vectors, as described in the Text S3 in Supporting Information S1.

### 3.2 Training Data

The AntSMB and Medley et al. (2014) data were modified to represent the relative deviation in snow accumulation from the large‐scale MERRA‐2 mean annual *P* − *E* (i.e., the percent deviation from MERRA‐2), which we hereinafter refer to as the SSV:

(1) $$\begin{array}{c} SSV=\frac{Observation-MERRA2}{\left|MERRA2\right|}\times 100\;. \end{array}$$

Because most observations are from GPR analysis, we generate two subsets: GPR and traditional, the latter includes stake, core, and snow pit measurements. Each set was then gridded onto the same 1‐km grid as the DEM by averaging points that fall within the same grid cell (Figure S2 in Supporting Information S1). As done with the DEM, we applied a 6‐km low‐pass filter.

The fact that this work used measurements from a large compilation of observations from a variety of techniques, means that there was not consistency in the temporal reference window across all observations. Thus, observations represented anywhere from a minimum of 3 years to over one thousand years, some of which overlapped with the MERRA‐2 time window and some of which did not, introducing additional uncertainty. While not ideal, we used all observations from all time windows to maximize the number of observations across as many conditions as possible; however, the bias introduced from a non‐coincident model and observation time window could have been propagated throughout our results.

### 3.3 Random Forest Method

Using the RF regression algorithm we predicted SSV over the entire AIS using 11 predictors (Section 3.1) and 2 training data sets (Section 3.2). The GPR (*n* = 27,316) gridded data were randomly sampled into 80% training and 20% testing partitions. We reserved an entire stake transect (*n* = 581) from the traditional data set to act as an independent model evaluation, and the remaining 2,535 traditional gridded values were split 80/20. Thus, a total of 23,881 observations were used for RF training. The testing partition was not used to build the RF model but rather for performance evaluation. Specifically, we employed bootstrap aggregation method (i.e., bagging) and an interaction‐based predictor‐selection technique for all RF experiments to increase detection of predictor interactions (Loh, 2002). The ensemble bagging technique builds decision trees each generated from a random sample with replacement of the training data set, diversifying the individual trees. The training data were weighted by the mean distance to all other observations, giving higher weights to those with more distant neighbors; this scheme minimizes the impact of GPR oversampling regions like West Antarctica.

Using two RF parameter scenarios (optimized and standard practice; Text S4 in Supporting Information S1), we built two final RF of 200 decision trees for SSV prediction using our ICESat‐2 DEM. The standard deviation amongst the individual trees provided an assessment of the spread in the prediction at the cell‐by‐cell basis, each of which were combined with the RMSE of the testing set (8.7%–9.0% depending on RF model; Figure S14 and Table S2 in Supporting Information S1) through root sum of squares to generate uncertainty, which is typically lower the closer the proximity to training observations. To investigate the impact of the choice of DEM, we employed the same exercise outlined above using the REMA DEM resampled to the same 1‐km grid as our ICESat‐2 DEM. Two CryoSat‐2 DEMs were not used (see Text S1.2 and S2.1 in Supporting Information S1). Thus, we built four SSV models.

## 4 Results

### 4.1 Small‐Scale Variability Predictions

**Figure 1:**
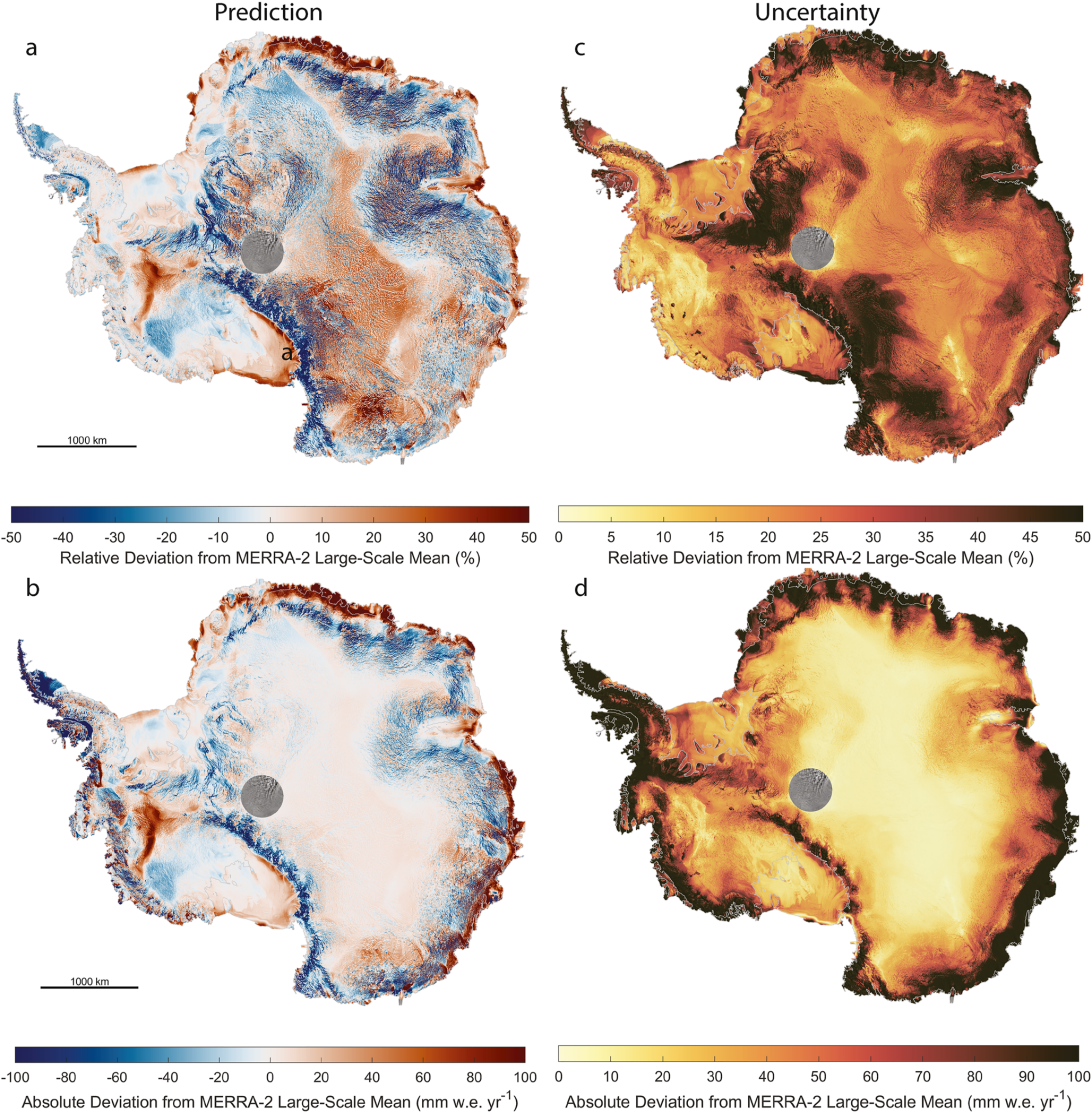
Predicted small‐scale variability (SSV) from the large‐scale mean Modern‐Era Retrospective analysis for Research and Applications, Version 2 accumulation and associated uncertainty. The (a) relative and (b) absolute predicted SSV show heterogenous patterns of deposition/erosion as well as larger‐scale model biases.

The SSV map provides insight into both the kilometer‐scale variability as well as the large‐scale biases in MERRA‐2 accumulation. Typical SSV (Figure 1a) range between −40.8% and +32.5% (lower and upper 5%), whereas absolute deviations in SSV (Figure 1b) span −63.2 to +64.2 mm w.e. yr^−1^. The largest SSV is −172%. Similarly, the SSV uncertainties (Figure 1c) range between 14.9% and 59.7%, whereas the absolute uncertainties (Figure 1d) span 5.3–201.4 mm w.e. yr^−1^. The uncertainties are larger in locations that are further from observations (Figure 1c). The RF models are strongly correlated with each other (all combinations *r* ^*2*^ > 0.94). When integrated in space, they predict between a reduction of 23.3 Gt yr^−1^ and an increase of 3.3 Gt yr^−1^ in the MERRA‐2 net accumulation (Table S2 in Supporting Information S1). Predictions on ice shelves suggest a more positive accumulation (+12.1 to +22.4 Gt yr^−1^). All indicate small to moderate reductions in the MERRA‐2 accumulation over grounded ice (−18.8 to −35.4 Gt yr^−1^).

Uncertainty calculations for the integrated values account for correlated errors within a 20‐km radius, a value chosen to correspond with the 20‐km high‐pass filtered surface heights used as a predictor. Because no model outperformed the others (Table S2 in Supporting Information S1), we present the most likely representation of SSV as the mean of all four predictions; we conservatively combine their cell‐by‐cell uncertainties through the root sum of squares. This approach yields integrated SSV for floating and grounded ice of +17.3 ± 11.7 and −25.0 ± 16.4 Gt yr^−1^, combining to −7.7 ± 20.1 Gt yr^−1^. Hereinafter, all results presented are in reference to this scenario. We note that the signal‐to‐noise ratio is >1 for only 11% of the ice sheet, indicating the uncertainty outweighs the signal (Figure S15 in Supporting Information S1). When comparing the RF model and its uncertainty bounds with the independent stake transect, however, we find the uncertainties are predominantly inflated (Figure S16 in Supporting Information S1). Specifically, the RMSE between the observed and modeled SSVs over the independent transect is 23% (Table S2 in Supporting Information S1), but the mean RF uncertainty amounts to 31%.

### 4.2 Comparison With ICESat‐2 Height Change

#### 4.2.1 Case Study With Coincident Snow Radar

Over long timescales and an unchanging climate, the amount of snow that falls and accumulates is balanced by firn compaction and the loss of firn via conversion to ice suggesting that ice‐surface‐height does not evolve because of snowfall processes; however, at sub annual scales, episodic and seasonal evolution of precipitation and temperature have a large impact on surface‐height changes. Thus, if our static SSV model is stable in time, then we should observe height changes that resemble the variability in snow accumulation. To investigate the importance of this variability on our interpretation of ice‐surface‐height evolution, we analyze the relationship between ICESat‐2 observed changes with our SSV model, Operation IceBridge (OIB) snow radar data, and MERRA‐2 climate.

**Figure 2:**
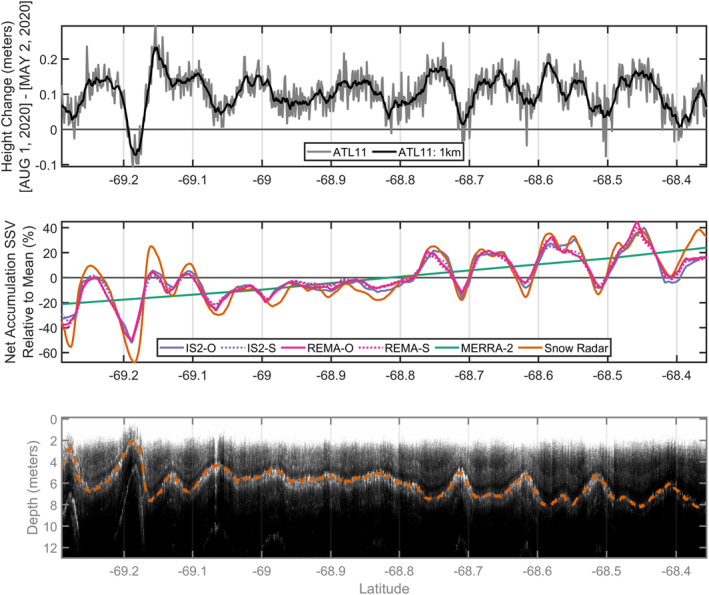
Comparison of Ice, Cloud, and land Elevation satellite (ICESat‐2) ATL11 height change with the random forest (RF) models of small‐scale variability (SSV) and radar‐derived snow accumulation. (a) The wintertime change in height (2 May 2020–1 August 2020) over a 100‐km ICESat‐2 ground track posted at 60 m (gray) and with a 1‐km moving average applied (black). (b) Snow accumulation relative to the large‐scale mean from Modern‐Era Retrospective analysis for Research and Applications, Version 2 (green), the four RF SSV models (pink/purple) named by the DEM used and whether the model used optimized (O) or standard (S) practice parameters, and coincident Operation IceBridge (OIB) snow radar‐derived snow accumulation. (c) OIB snow radar echogram collected 25 October 2019 that is coincident in space with the ICESat‐2 ATL11 reference pair track 2. The layer traced in dashed orange provided the basis of the radar‐derived snow accumulation represented by an orange line in panel (b). This snow radar transect is mapped in Figure S1b in Supporting Information S1.

In 2019, OIB underflew ICESat‐2 ground tracks over coastal Wilkes and Victoria Land, which provides us the ability to directly compare OIB snow radar, our SSV models, and ICESat‐2 height change. We analyze a 100‐km segment from 25 October 2019 that follows a trajectory near‐perpendicular to the coast (Figure 2 and Figure S1b in Supporting Information S1). The ICESat‐2 height change along this ground track between 2 May 2020 and 1 August 2020 shows an overall increase with significant small‐scale variations along track (Figure 2a). We next compare the ICESat‐2 data with coincident OIB snow radar data by following the same procedure as outlined by Dattler et al. (2019) to produce net accumulation by tracking a single radar horizon through space. We show the resulting radar‐derived accumulation, the SSV models, and the MERRA‐2 mean *P* − *E* interpolated to each radar measurement in Figure 2b, and the snow radar echogram and tracked layer in Figure 2c. As with the Dattler et al. (2019) data set, our radar‐derived accumulations were calculated in a way that matches them with the large‐scale MERRA‐2 mean. That assumption does not impact our assessment of the SSV in the snow accumulation.

Based on this exercise, we confirm that our models can predict SSV in snow accumulation and that there are not substantial differences between the RF models. We note that these snow radar data were collected in 2019 and are not part of the GPR accumulation data set compiled by Dattler et al. (2019), which used data collected up through 2017. Thus, the comparison here is independent of our RF model development. We also confirm that the RF models underestimate the total magnitude of the larger deviations. Nevertheless, we observe significant correlation between the radar‐derived accumulations, RF models of SSV, and ICESat‐2 height change variability.

### 4.3 Ice‐Sheet‐Wide Height Change and Snow Accumulation Variability

**Figure 3:**
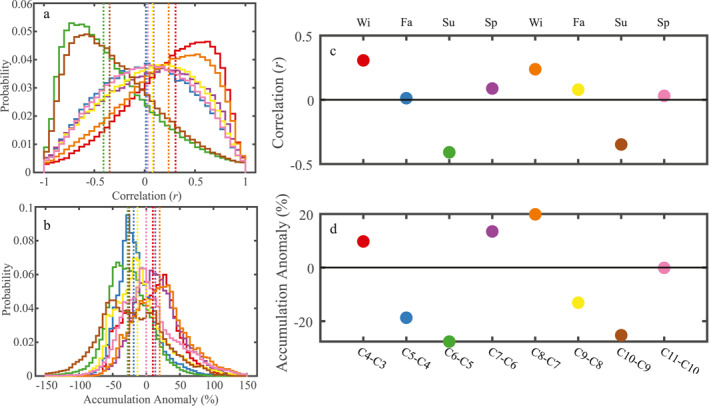
Comparison of 50‐km along‐track (a) correlations between the mean random forest net accumulation model and Ice, Cloud, and land Elevation satellite ATL11 height change for eight cycle pairs and (b) temporal snow accumulation anomalies over the entire Antarctic Ice Sheet. The results are presented as histograms of either the correlation coefficient or the magnitude of the temporal anomaly in snow accumulation over 50‐km ATL11 segments and are color coded by cycle pair. The median of each distribution is displayed as a dotted vertical line. (c) The median correlation coefficient values from (a) plotted in time referenced to the cycle pair and its associated season. (d) The same as (c) but a time series of the median accumulation anomaly. Colors in both panels (c and d) match those from panels (a and b).

ICESat‐2 ATL11 provides along‐track, slope‐corrected heights spanning nine 91‐day cycles, providing seasonal height change over a 2‐year period. For each reference pair track, we calculate cycle‐by‐cycle height change (i.e., height change over a 91‐day interval) and apply a 6‐km moving mean to match the same filter applied to our ICESat‐2 DEM (Text S2 in Supporting Information S1). For each reference pair track, we find the mean MERRA‐2 *P* − *E* anomaly over the exact time epoch for each cycle and for that specific track. This step provides the temporal accumulation anomaly along each reference pair track over the same time and space as the ICESat‐2 ATL11 data. We also interpolate our static RF SSV models onto the ATL11 heights. To investigate the relationship between observed height change and our predicted SSV, we correlate the ATL11 height changes with the mean predicted SSV along 50‐km segments for each cycle pair. This relationship, as well as the temporal accumulation anomaly over each cycle pair, is summarized in Figure 3. We find that the sign and magnitude of the correspondence between spatial variations in snow accumulation and observed height changes varies by season.

## 5 Discussion

We use a combination of snow accumulation derived from GPR, as well as other traditional observational constraints, with topographic and atmospheric characteristics derived from ICESat‐2 surface height data and MERRA‐2 to predict net accumulation on a 1‐km grid. Neither selection of the RF model parameters nor choice of DEM largely impacted the results, suggesting that we used a robust choice of predictors. Comparison of performance statistics on the testing and training data sets suggest some RF model overfitting given the increased performance of the training data set; however, the models remain performant at a level similar to the statistics for the testing and transect subsets in unobserved regions, and the uncertainties at those locations reflect the reduced performance. Not all predictors, however, were equally important. We found that MSWD was by far the most influential predictor followed by wind speed, *P* − *E*, and wind direction in order.

Our new SSV predictions over the entire AIS suggest an insignificant reduction of 7.7 ± 20.1 Gt yr^−1^, which means there is no significant difference from the integrated MERRA‐2 large‐scale mean; however, our map shows substantial deviations at the regional to local scales that are indicative of increased net accumulation over the ice shelves (+17.3 ± 11.7 Gt yr^−1^) and decreased net accumulation over the grounded ice sheet (−25.0 ± 16.4 Gt yr^−1^). Thus, we find that while MERRA‐2 provides realistic estimates of integrated accumulation, locally it fails to capture the local‐to‐regional deviations, which is unsurprising as the global model cannot resolve finer‐scale topography.

### 5.1 Snow Accumulation Variability and Height Change

At seasonal time scales, variations in surface height fluctuate in response to strong positive or negative snowfall anomalies in time, albeit in a different fashion. Over the entire AIS, an integrated positive anomaly (Figure 3: red/orange) typically occurs in winter, when the SSV model is positively correlated with observed height changes. Locations that receive higher net accumulation than its immediate vicinity experience larger height increases, which have not yet been modulated by their enhanced compaction rates, which operate on slower timescales. The opposite is true in the summer when the ice sheet typically experiences negative accumulation anomalies (Figure 3: green/brown): locations that receive anomalously higher net accumulation than its immediate vicinity experience larger height decreases. Even though a region might not receive any accumulation, densification processes are more rapid where the long‐term net accumulation is larger; thus, under anomalously low accumulation conditions, we observe the spatial variations in compaction rates that are generated from the spatial variations in the long‐term net accumulation.

The signal when integrated over the entire ice sheet is less obvious in spring and summer. We hypothesize that while during spring (Figure 3: purple/pink) there are typically large negative anomalies in accumulation, the firn column remains cold coming out of winter, which reduces compaction rates and thus the correspondence between the SSV and ATL11 height changes. We expect the opposite as well: during the fall (Figure 3: blue/yellow), the firn column is warmer leading to more compaction, which counterbalances the typical positive snow accumulation anomalies, although the signal is weaker.

These results indicate that substantial deviations in ATL11 height changes along‐track exist in response to small‐scale variations in the net accumulation and that the sign of the height change anomaly likely reflects the sign of the temporal accumulation anomaly over the cycle‐pair epoch. Thus, variability observed in ATL11 derived height change reflect surface processes and should not be considered instrument noise but rather highlight precision and data product capability. Thus, any studies interested in change over short length scales will need to strongly consider the impact of surface processes on the interpretation of the observed spatiotemporal height changes.

### 5.2 Limitations

While we have provided a product of AIS net snow accumulation that is largely capable of reproducing its spatial variability, several limitations remain that if addressed could improve the methodology. In the generation of the DEM, we chose to remove any ATL06 surface heights that had an RMS error larger than 0.1 m, which likely excluded too much data in steeply sloping regions. This limitation could be overcome using an RMS threshold as a function of slope. Because the technique used to derive the OIB snow accumulation is tied to the MERRA‐2 large‐scale mean (Dattler et al., 2019), we only use MERRA‐2 atmospheric data as predictors. Given that the RF models predict accumulation variability due to small‐scale topographic deviations as well as to large‐scale biases in MERRA‐2, we cannot disentangle the two from one another, making it is difficult to attribute their individual contributions.

Other limitations stem from the predictor training data used. While the topographic data are well resolved at 1‐km resolution, the atmospheric data only resolves variables at several 10s of km; thus, atmospheric downscaling could lead to improved predictions. The set of predictors used might also be incomplete. Our analysis suggests that height change from ICESat‐2 is also strongly related to the SSV in snow accumulation, and it could provide more constraint in the future at the ice‐sheet‐wide scale. Similarly, the RF model relies on training data spanning several different atmospheric and topographic regimes, however, most of the GPR observations are from the Antarctic Peninsula and West Antarctica. The traditional data set fills in much of the missing areas in East Antarctica, but much of the data are representative of a single point, which might not be representative of the 1 km‐by‐1 km region in which it falls.

## 6 Conclusions

While atmospheric models generally agree on the synoptic‐scale signatures of snow accumulation over the AIS (Mottram et al., 2021), they at present either do not account for drifting snow processes or do so at a coarse scale. Shallow radar studies have revealed significant deviations in the snow accumulation at sub‐grid‐cell scales (Medley et al., 2013; Richardson et al., 1997; Spikes et al., 2004), which suggest that atmospheric model evaluations against sparse point measurements of snow accumulation are likely flawed. The predictions generated for this study will hopefully provide new context for model evaluations by eliminating some of the scale ambiguity in model‐observation comparisons. The resulting spatial anomalies in the net accumulation are manifested in satellite‐derived measurements of surface height changes, which also adds uncertainty to interpretation especially when considering seasonal timescales. Additional measurements of the small‐scale variations in snow accumulation as well as more targeted studies bringing together satellite altimetric height changes and firn densification models at the local scale would prove more edifying in untangling the full response of the surface to these various processes.

## Supporting information

Supporting Information S1

## Data Availability

The ICESat‐2 data used in this study are available in Smith et al. (2019, 2021). The MERRA‐2 data used are in GMAO (2015a, 2015b, 2015c, 2015d). The AntSMB data are available at https://doi.org/10.11888/Glacio.tpdc.271148. The IceBridge snow radar data are available in Paden et al. (2014), and the IceBridge Airborne Topographic Mapper data are available in Studinger (2014). The Reference Elevation Model of Antarctica is available in Howat et al. (2019), and the CryoSat‐2 DEMs are available in Helm et al. (2014a, 2014b) and Slater et al. (2018). The data created in this study, as well as the Medley et al. (2014) radar‐derived snow accumulation data, are available at https://doi.org/10.5281/zenodo.7105855.

## References

[grl64917-bib-0001] Agosta, C. , Amory, C. , Kittel, C. , Orsi, A. , Favier, V. , Gallée, H. , et al. (2019). Estimation of the Antarctic surface mass balance using the regional climate model MAR (1979‐2015) and identification of dominant processes. The Cryosphere, 13(1), 281–296. 10.5194/tc-13-281-2019

[grl64917-bib-0002] Amory, C. , Kittel, C. , Le Toumelin, L. , Agosta, C. , Delhasse, A. , Favier, V. , & Fettweis, X. (2021). Performance of MAR (v3.11) in simulating the drifting‐snow climate and surface mass balance of Adélie Land, East Antarctica. Geoscientific Model Development, 14(6), 3487–3510. 10.5194/gmd-14-3487-2021

[grl64917-bib-0003] Das, I. , Bell, R. E. , Scambos, T. A. , Wolovick, M. , Creyts, T. T. , Studinger, M. , et al. (2013). Influence of persistent wind scour on the surface mass balance of Antarctica. Nature Geoscience, 6(5), 367–371. 10.1038/ngeo1766

[grl64917-bib-0004] Dattler, M. E. , Lenaerts, J. T. M. , & Medley, B. (2019). Significant spatial variability in radar‐derived west Antarctic accumulation linked to surface winds and topography. Geophysical Research Letters, 46(22), 13126–13134. 10.1029/2019GL085363

[grl64917-bib-0005] Gelaro, R. , McCarty, W. , Suárez, M. J. , Todling, R. , Molod, A. , Takacs, L. , et al. (2017). The modern‐era retrospective analysis for research and applications, version 2 (MERRA‐2). Journal of Climate, 30(14), 5419–5454. 10.1175/JCLI-D-16-0758.1 PMC6999672 32020988

[grl64917-bib-0006] GMAO . (2015a). MERRA‐2 tavg1_2d_flx_Nx: 2d, 1‐hourly, time‐averaged, single‐level, assimilation, surface flux diagnostics V5.12.4 [Dataset]. NASA Goddard Earth Sciences Data and Information Services Center. 10.5067/7MCPBJ41Y0K6

[grl64917-bib-0007] GMAO . (2015b). MERRA‐2 tavg1_2d_slv_Nx: 2d, 1‐hourly, time‐averaged, single‐level, assimilation, single‐level diagnostics V5.12.4 [Dataset]. NASA Goddard Earth Sciences Data and Information Services Center. 10.5067/VJAFPLI1CSIV

[grl64917-bib-0008] GMAO . (2015c). MERRA‐2 tavgM_2d_flx_Nx: 2d, monthly mean, time‐averaged, single‐level, assimilation, surface flux diagnostics V5.12.4 [Dataset]. NASA Goddard Earth Sciences Data and Information Services Center. 10.5067/0JRLVL8YV2Y4

[grl64917-bib-0009] GMAO . (2015d). MERRA‐2 tavgM_2d_slv_Nx: 2d, monthly mean, time‐averaged, single‐level, assimilation, single‐level diagnostics V5.12.4 [Dataset]. NASA Goddard Earth Sciences Data and Information Services Center. 10.5067/AP1B0BA5PD2K

[grl64917-bib-0010] Helm, V. , Humbert, A. , & Miller, H. (2014a). Elevation and elevation change of Greenland and Antarctica derived from CryoSat‐2. The Cryosphere, 8(4), 1539–1559. 10.5194/tc-8-1539-2014

[grl64917-bib-0011] Helm, V. , Humbert, A. , & Miller, H. (2014b). Elevation model of Antarctica derived from CryoSat‐2 in the period 2011 to 2013, links to DEM and uncertainty map as GeoTIFF [Dataset]. Pangaea. 10.1594/PANGAEA.831392

[grl64917-bib-0012] Howat, I. M. , Porter, C. , Smith, B. E. , Noh, M.‐J. , & Morin, P. (2019). The reference elevation model of Antarctica. The Cryosphere, 13(2), 665–674. 10.5194/tc-13-665-2019

[grl64917-bib-0014] Lenaerts, J. T. , Ligtenberg, S. R. , Medley, B. , Van de Berg, W. J. , Konrad, H. , Nicolas, J. P. , et al. (2018). Climate and surface mass balance of coastal West Antarctica resolved by regional climate modelling. Annals of Glaciology, 59(76pt1), 29–41. 10.1017/aog.2017.42

[grl64917-bib-0015] Lenaerts, J. T. , Medley, B. , van den Broeke, M. R. , & Wouters, B. (2019). Observing and modeling ice sheet surface mass balance. Reviews of Geophysics, 57(2), 376–420. 10.1029/2018RG000622 31598609 PMC6774314

[grl64917-bib-0016] Lenaerts, J. T. , Van den Broeke, M. R. , Van de Berg, W. J. , Van Meijgaard, E. , & Kuipers Munneke, P. (2012). A new, high‐resolution surface mass balance map of Antarctica (1979–2010) based on regional atmospheric climate modeling. Geophysical Research Letters, 39(4), L04501. 10.1029/2011GL050713

[grl64917-bib-0018] Loh, W.‐Y. (2002). Regression tress with unbiased variable selection and interaction detection. Statistica Sinica, 12(2), 361–386. Retrieved from http://www.jstor.org/stable/24306967

[grl64917-bib-0019] Markus, T. , Neumann, T. , Martino, A. , Abdalati, W. , Brunt, K. , Csatho, B. , et al. (2017). The Ice, Cloud, and land Elevation satellite‐2 (ICESat‐2): Science requirements, concept, and implementation. Remote Sensing of Environment, 190, 260–273. 10.1016/j.rse.2016.12.029

[grl64917-bib-0020] Medley, B. , Joughin, I. , Das, S. B. , Steig, E. J. , Conway, H. , Gogineni, S. , et al. (2013). Airborne‐radar and ice‐core observations of annual snow accumulation over Thwaites Glacier, West Antarctica confirm the spatiotemporal variability of global and regional atmospheric models. Geophysical Research Letters, 40(14), 3649–3654. 10.1002/grl.50706

[grl64917-bib-0021] Medley, B. , Joughin, I. , Smith, B. E. , Das, S. B. , Steig, E. J. , Conway, H. , et al. (2014). Constraining the recent mass balance of Pine Island and Thwaites glaciers, West Antarctica, with airborne observations of snow accumulation. The Cryosphere, 8(4), 1375–1392. 10.5194/tc-8-1375-2014

[grl64917-bib-0022] Mottram, R. , Hansen, N. , Kittel, C. , van Wessem, J. M. , Agosta, C. , Amory, C. , et al. (2021). What is the surface mass balance of Antarctica? An intercomparison of regional climate model estimates. The Cryosphere, 15(8), 3751–3784. 10.5194/tc-15-3751-2021

[grl64917-bib-0036] Paden, J. , Li, J. , Leuschen, C. , Rodriguez‐Morales, F. , & Hale, R. (2014). IceBridge snow radar L1B geolocated radar echo strength profiles (Version 2) [Dataset]. NASA National Snow and Ice Data Center Distributed Active Archive Center. 10.5067/FAZTWP500V70

[grl64917-bib-0023] Richardson, C. , Aarholt, E. , Hamran, S.‐E. , Holmlund, P. , & Isaksson, E. (1997). Spatial distribution of snow in western Dronning Maud Land, East Antarctica, mapped by a ground‐based snow radar. Journal of Geophysical Research, 102(B9), 20343–20353. 10.1029/97JB01441

[grl64917-bib-0024] Rignot, E. , Mouginot, J. , Scheuchl, B. , Van Den Broeke, M. , Van Wessem, M. J. , & Morlighem, M. (2019). Four decades of Antarctic Ice Sheet mass balance from 1979–2017. Proceedings of the National Academy of Sciences of the United States of America, 116(4), 1095–1103. 10.1073/pnas.1812883116 30642972 PMC6347714

[grl64917-bib-0025] Scambos, T. A. , Frezzotti, M. , Haran, T. , Bohlander, J. , Lenaerts, J. T. M. , Van Den Broeke, M. R. , et al. (2012). Extent of low‐accumulation “wind glaze” areas on the East Antarctic plateau: Implications for continental ice mass balance. Journal of Glaciology, 58(210), 633–647. 10.3189/2012JoG11J232

[grl64917-bib-0026] Shepherd, A. , Ivins, E. , Rignot, E. , Smith, B. , Van Den Broeke, M. , Velicogna, I. , et al. (2018). Mass balance of the Antarctic Ice Sheet from 1992 to 2017. Nature, 558(7709), 219–222. 10.1038/s41586-018-0179-y 29899482

[grl64917-bib-0027] Slater, T. , Shepherd, A. , McMillan, M. , Muir, A. , Gilbert, L. , Hogg, A. E. , et al. (2018). A new digital elevation model of Antarctica derived from CryoSat‐2 altimetry. The Cryosphere, 12(4), 1551–1562. 10.5194/tc-12-1551-2018

[grl64917-bib-0028] Smith, B. , Dickinson, S. , Jelley, B. P. , Neumann, T. A. , Hancock, D. , Lee, J. , & Harbeck, K. (2021). ATLAS/ICESat‐2 L3B annual land ice height, version 4 [Dataset]. NASA National Snow and Ice Data Center DAAC. 10.5067/ATLAS/ATL11.004

[grl64917-bib-0029] Smith, B. , Fricker, H. A. , Gardner, A. , Siegfried, M. R. , Adusumilli, S. , Csatho, B. M. , et al. (2019). ATLAS/ICESat‐2 L3A land ice height, version 2 [Dataset]. NASA National Snow and Ice Data Center DAAC. 10.5067/ATLAS/ATL06.002

[grl64917-bib-0030] Smith, B. , Fricker, H. A. , Gardner, A. S. , Medley, B. , Nilsson, J. , Paolo, F. S. , et al. (2020). Pervasive ice sheet mass loss reflects competing ocean and atmosphere processes. Science, 368(6496), 1239–1242. 10.1126/science.aaz5845 32354841

[grl64917-bib-0031] Spikes, V. B. , Hamilton, G. S. , Arcone, S. A. , Kaspari, S. , & Mayewski, P. A. (2004). Variability in accumulation rates from GPR profiling on the West Antarctic plateau. Annals of Glaciology, 39, 238–244. 10.3189/172756404781814393

[grl64917-bib-0013] Studinger, M. (2014). IceBridge ATM L2 Icessn elevation, slope, and roughness, version 2 [Dataset]. NASA National Snow and Ice Data Center DAAC. 10.5067/CPRXXK3F39RV

[grl64917-bib-0032] Studinger, M. , Medley, B. C. , Brunt, K. M. , Casey, K. A. , Kurtz, N. T. , Manizade, S. S. , et al. (2020). Temporal and spatial variability in surface roughness and accumulation rate around 88°S from repeat airborne geophysical surveys. The Cryosphere, 14(10), 3287–3308. 10.5194/tc-14-3287-2020

[grl64917-bib-0033] Van Wessem, J. M. , Jan Van De Berg, W. , Noël, B. P. , Van Meijgaard, E. , Amory, C. , Birnbaum, G. , et al. (2018). Modelling the climate and surface mass balance of polar ice sheets using RACMO2: Part 2: Antarctica (1979‐2016). The Cryosphere, 12(4), 1479–1498. 10.5194/tc-12-1479-2018

[grl64917-bib-0034] Wang, Y. , Ding, M. , Reijmer, C. H. , Smeets, P. C. , Hou, S. , & Xiao, C. (2021). The AntSMB dataset: A comprehensive compilation of surface mass balance field observations over the Antarctic Ice Sheet. Earth System Science Data, 13(6), 3057–3074. 10.5194/essd-13-3057-2021

