# Supplementary material for: Predicting Antarctic Net Snow Accumulation at the Kilometer Scale and Its Impact on Observed Height Changes
Source: Geophys Res Lett. 2022 Oct 17;49(20):e2022GL099330. doi: 10.1029/2022GL099330 (PMC9787652; doi:10.1029/2022GL099330)
Supplement: Supplementary file 1 — Supporting Information S1 [file GRL-49-e2022GL099330-s001.docx]

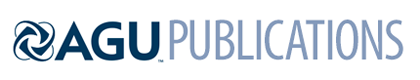


*Geophysical Research Letters*

Supporting Information for

**Predicting Antarctic net snow accumulation at the kilometer scale and its impact on observed height changes**

B. Medley^1^, J. T. M. Lenaerts^2^, M. Dattler^1,3^, E. Keenan^2^, and N. Wever^2^

^1^Cryospheric Sciences Laboratory, NASA Goddard Space Flight Center, Greenbelt, MD, USA.

^2^Department of Atmospheric and Oceanic Sciences, University of Colorado Boulder, Boulder, CO, USA.

^3^Department of Atmospheric and Oceanic Science, University of Maryland College Park, College Park, MD, USA.

**Contents of this file**

Text S1 to S5

Figures S1 to S16

Tables S1 to S2

**Introduction**

This supplement includes details regarding the digital elevation models used with specific details on how the ICESat-2 DEM was generated and evaluated. We also explain the creation of the random forest model predictor of the mean slope in the weighted mean wind direction. Next, we describe in detail the specifics behind our random forest modeling of snow accumulation variability, and we expand on the results by region. Finally, we provide maps of each of the predictors used in our random forest model.

Access to the data generated in this work are available at <https://doi.org/10.5281/zenodo.7105855>.

Text S1. Supplemental Data

S1.1 Airborne Topographic Mapper

NASA’s Airborne Topographic Mapper (ATM) is a scanning airborne laser altimeter and was one of several instruments used during Operation IceBridge’s (OIB) annual airborne surveys over Antarctica, beginning in 2009. Here, we use the Icessn Elevation, Slope, and Roughness, Version 2 (ILATM2; Studinger (2014)) data product collected between 2009 and 2018. OIB typically surveyed the Antarctic Ice Sheet between mid-October to mid-November, and ATM was flown each year with the exception of 2015, providing 9 years of surface heights over much of West Antarctica and the Antarctic Peninsula and smaller portions of East Antarctica. The ATM ILATM2 data are used as an independent assessment of Digital Elevation Model (DEM) quality.

S1.2 Digital Elevation Models

We use three additional digital elevation models (DEMs) to evaluate the importance of accurate height measurements for understanding snow redistribution. Two of the DEMs were derived using ESA’s CryoSat-2 radar altimeter data (Helm et al., 2014; Slater et al., 2018), and the third was generated using commercial-satellite high-resolution stereophotogrammetry, referred to as the Reference Elevation Model of Antarctica (REMA; Howat et al., 2019). The CryoSat-2 DEMs are posted at 1 km resolution, however, when operating in the Low Resolution Mode, CryoSat-2’s footprint is larger than 1 km^2^. We use the REMA 200 m mosaic, however, REMA is available at several resolutions.

Text S2. ICESat-2 DEM Generation

We incrementally built an Antarctic-wide DEM based on ICESat-2 surface height retrievals in 50 km-by-50 km tiles. For each tile, we used ATL06 data that fell within the tile and a 3 km-wide buffer around its periphery. ATL06 retrievals that had Root Mean Square (RMS) solutions larger than 0.1 m were excluded from the analysis. Heights outside of 5σ (standard deviations) of the mean height within the tile were also excluded, which predominantly removed regions that were likely impacted by clouds. To remove small-scale noise, we similarly removed height retrievals outside of 5σ within a 2 km search radius. Within each grid cell, we then assessed the mean, median, standard deviation, and count of valid ATL06 surface height retrievals. If no height retrievals existed within a grid cell, it remained empty. This process was applied to grids of 250 m, 500 m, 1 km, and 2 km, producing four DEMs of variable resolution for each tile.

The 5,695 50 km-by-50 km tiles were then combined to a master 1-km DEM (Figure S1). We investigated the potential for bad ATL06 granules by accumulating the number of grid cells with standard errors greater than 3σ of the Antarctic-wide mean that intersect the ground tracks within each granule. For those granules with a count of flagged cells greater than 3σ of the mean, we determined the RMS deviation from REMA. Out of the 10,898 granules used, we flagged only five as problematic based on the fact that their deviations from REMA were an order of magnitude larger than the others. These problematic granules were removed, and the tiles that contained the potential bad data were regenerated to complete the final master DEM.

For this study, we built a 1-km DEM; however, to assist in filling gaps in the northern portions, we populate any empty 1-km grid cells with data from an underlying 2-km grid cell, if it exists. In all, 91.4% of the 1-km grid cells contained ATL06 surface height retrievals, and only 2.0% of the cells were filled using the underlying 2-km grid. The remaining 6.7% of cells were filled with modified REMA surface heights. Because the data used to generate REMA were not contemporaneous with the ICESat-2 data, we must correct the REMA heights to equivalent ICESat-2 heights. For each empty 1-km cell, we extracted all existing 1-km ICESat-2 grid cells within 25 km of the empty cell, along with the equivalent REMA heights, to build a linear regression to predict the ICESat-2 equivalent of the REMA height. The 25-km window was widened iteratively until there were at least 200 points for building the regression model. The error associated with the REMA-filled cells is the 99% confidence interval for the regression model.

S2.1 Digital Elevation Model Skill

We gridded the ATM ILATM2 data onto the same 1-km grid as the ICESat-2 DEM, averaging points that fall within the same grid cell (Figure S3). These 196,406 measurements collected over 9 years provide an independent assessment of several DEMs used in this study. We evaluated the DEMs using annual grids of individual OIB campaign years as well as a single grid built using the data from the annual grids that represent the most recent OIB campaign year on a grid cell by grid cell basis. The latter maximizes the use of near-contemporaneous data because ICESat-2 launched towards the end of OIB in late 2018. Table S1 contains summary statistics for ATM evaluation of the DEMs. We found that our ICESat-2 DEM is of comparable quality as the REMA DEM and of better quality than the two CryoSat-2 DEMs. Because of their ability to accurately reproduce the ATM ILATM2 measurements, we elected to use only the ICESat-2 and REMA DEMs to produce snow redistribution estimates.

Text S3. Weighting Wind Conditions by Snowfall

One commonly used predictor for snow erosion over the Antarctic Ice Sheet is the mean slope in the mean wind direction (MSWD), which combines the wind direction and surface slope via their dot product (Das et al., 2012; Dattler et al., 2019; Scambos et al., 2012; Studinger et al., 2020). The mean annual wind vectors were typically used for this proxy; however, wind speed and orientation is often largely different under precipitating conditions when the new snow is most susceptible to redistribution (Lacroix et al., 2009; Trujillo et al., 2016). Here, we investigate modifications to the MSWD to better replicate conditions where snow erosion/deposition might occur.

In addition to using the MSWD, we used MERRA-2 hourly snowfall data to weight wind variables under two scenarios. For scenario 1, we determined for each hourly time step, *t*, the total snowfall accumulated *Δt* prior to *t*, for which we investigated choices of *Δt* ranging between 1 and 12 hours. We then determined the mean wind conditions when the accumulated snowfall was greater than zero for each *Δt*. This scenario provides the typical wind conditions when there is contemporaneous snowfall or snow has accumulated shortly prior. For scenario 2, we calculated a weighted mean of wind conditions based on the total snowfall accumulated over each *Δt* interval. Combining the MSWD and these two scenarios over the range of *Δt* yields 25 different sampling of mean wind conditions for each grid cell. We determined the optimal choice of scenario and *Δt* for investigating snow redistribution by evaluating their importance as a predictor (see Section 3). We found that scenario 1 and a *Δt* of 9 hours (i.e., mean wind conditions only when snowfall occurred within the past 9 hours) provided the best prediction; however, we note that predictor capability did not vary substantially by *Δt* and was only marginally better under scenario 1 than scenario 2.

Text S4. Random Forest Model

S4.1 Parameter Scenarios and Optimization

We next optimized three RF algorithm parameters: (1) the maximum number of splits and (2) minimum leaf size, both of which determine the tree depth, and (3) the number of predictors to sample at each split, which adds to randomness amongst the individual trees. We then evaluated several RF predictions, each with 100 trees, sampling a range of the three aforementioned parameters. Specifically, we investigated maximum splits between 1,000 and 5,000 (sampling interval of 200), minimum leaf size between 1 and 9 (sampling interval of 1), and number of predictors to sample between 3 and 11 (sampling interval of 1). The skill of each parameter combination was assessed using the coefficient of determination (*r^2^*), the root mean square error (*RMSE*), and the slope (*m*) between the test features and their predictions. Considering equal importance of the skill evaluators between the GPR and traditional test observations, we determined optimal choices of 4,600 maximum number of splits for the ICESat-2 DEM and 4,200 for the REMA DEM, a minimum leaf size of 1 for ICESat-2 and 2 for REMA, and number of predictors to sample of 6 for ICESat-2 and 4 for REMA. Although ensemble bagging reduces the chance of overfitting by randomizing decision trees via sub selection of training observations and predictors when splitting nodes, we also investigated using commonly used choices for the RF parameters: a minimum leaf size of 5, common for regression trees, and the number of predictors to sample equal to one-third the number of predictors (${11}/3\approx3)$.

S4.2 Model Skill

We evaluated the performance of the four RF scenarios using both out-of-box statistics and statistics derived through comparison with our independent testing partitions of the GPR and traditional snow accumulation measurements as well as an entire transect removed from the traditional dataset. Because the ensemble bagging technique builds decision trees each generated from a random sample with replacement of the training dataset, for a given decision tree, some of the training data will be sampled repeatedly while others will be left out: the latter comprises the out-of-box samples. The out-of-box error presented for each RF model is the mean squared error of the out-of-box predictions when compared to the actual observation. Table S2 summarizes the evaluations. We performed additional statistical tests of each RF using the training and testing partitions independently. The latter provides a more realistic evaluation of prediction, while the former provides context as the “best-case scenario” performance; however, the latter still is inadequate because these observations are largely correlated in space, and random selection of the testing dataset will likely yield overperformance when compared to locations that are far from observations. Thus, model performance against the testing dataset is likely overestimated, so we also examine model performance on an entirely unused transect (see location in Figure S1b). The RF predictions from the four models all have similar performance with similar predictive capability of the GPR and traditional observations (train and test). The RMSE is larger for the traditional measurements because the data are typically not smoothed since they are more isolated in space, which results in more extreme values than the GPR data. The mean RMSE of all four models when evaluating the GPR and traditional measurements together was 5.2% for the training data and 8.8% for the testing data. The prediction performance along the transect was slightly reduced when compared to the testing data, which suggests that the performance statistics over unsampled areas (or areas far from observations) are slightly degraded from the statistics derived from the testing dataset. At the same time, the differences are not necessarily substantial, which indicates that the model is still providing realistic predictions over unsampled areas.

The average coefficient of determination (*R*^2^) between the observed and predicted deviations for all four RF models was 0.91, 0.67, and 0.43 for the training, testing, and transect partitions, respectively (Table S2). These relationships suggest that the predictions are capturing much of the spatial variability in the accumulation deviations. Regression analysis of the observations and predictions, however, indicates that the RF models are potentially underpredicting the magnitude of these deviations (Figure S14). Specifically, the average slope between the observed and predicted deviations fall short of unity: 0.80, 0.59, and 0.51 for the training, testing, and transect partitions, respectively (Table S2). These evaluations are used in Section 5 to aid in interpretation of the results.

Text S5. Spatial Patterns

From an integrated mass balance perspective, we predict a reduction in net accumulation over the Antarctic Peninsula (AP; -25.0 Gt yr-1), a moderate increase over the East Antarctic Ice Sheet (EAIS; +14.6 Gt yr-1), and a small increase over the West Antarctic Ice Sheet (WAIS; +2.7 Gt yr-1). Because there are several observations from West Antarctica, the uncertainties in both an absolute and relative sense are quite low. Thus, any additional measurements from WAIS will likely not provide much improvement to our predictions. Interestingly, traverse measurements do exist within the sector spanning eastern Dronning Maud Land to the Amery Ice Shelf; however, the uncertainties here remain large, which could be a function of the very large SSV and perhaps a strongly different climate and/or topography as the region contributing the most measurements (i.e., WAIS).

The largest mass losses occur in eastern Dronning Maud Land and Wilkes Land; however, when the mass loss from redistribution is considered as a percentage of the total precipitation within that sector, eastern Dronning Maud Land, Enderby Land, Mac. Robertson Land, and the region surrounding the Amery Ice Shelf are the most impacted by snow redistribution losses followed by coastal Victoria Land. The scale of the variability in snow redistribution processes is also of interest when interpreting height changes through time or considering ice-core retrieval locations. The sectors with the largest typical deviations from the MERRA-2 large-scale mean are located in the central and eastern WAIS and over the AP, which is largely due to the very high snowfall amounts from these sectors (i.e., there is more snow available to redistribute or sublimate). The regions with the smallest deviations are western Dronning Maud Land and the Ross Sea Sector.

We also investigate the spatial patterns of the errors in our snow redistribution model to not only show the limitations of the model, but also highlight potential improvements. Considering a regional mean in the uncertainty in the relative snow redistribution, we find that the lowest uncertainties are focused over WAIS where the highest density of OIB observations exist whereas the highest uncertainties are over eastern Dronning Maud Land, Enderby Land, Mac. Robertson Land, and the region surrounding the Amery Ice Shelf. The largest absolute uncertainties are found over the AP whereas the smallest are found over the Marie Byrd Land and western Dronning Maud Land.


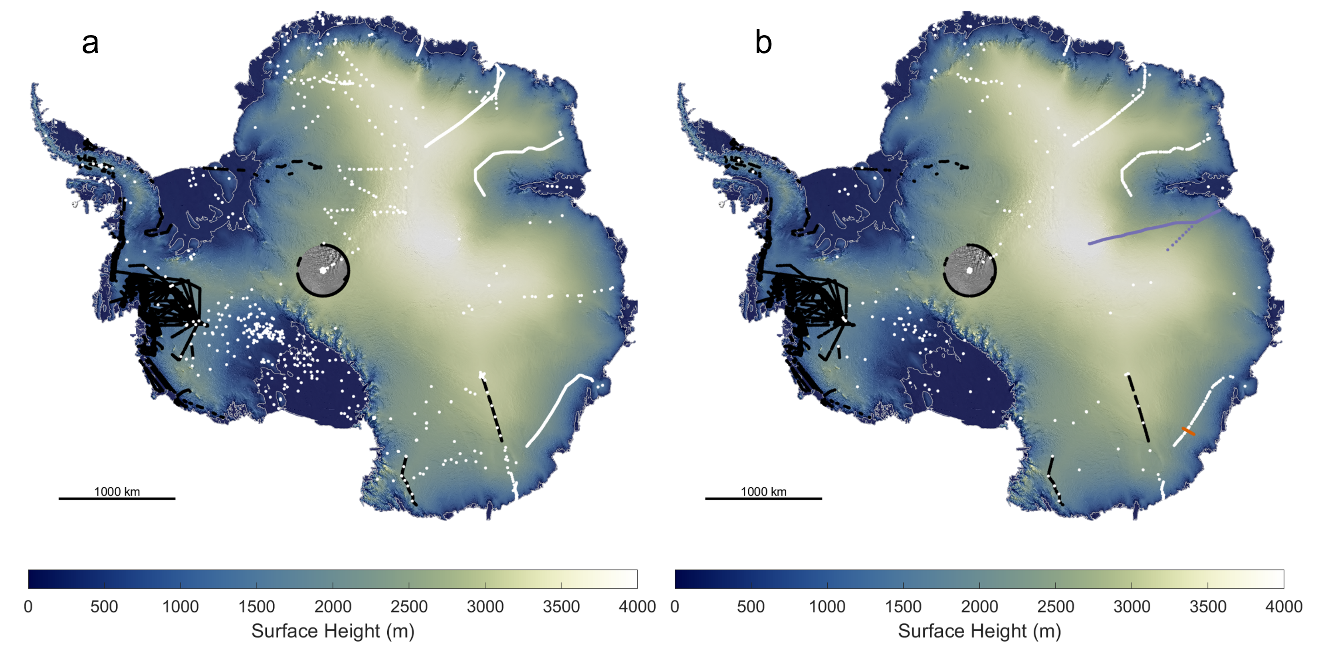


Figure S1. Locations of the snow accumulation rates used to (a) train and (b) test the RFs from GPR (black dots) and the Traditional dataset (white dots) overlaid on a 1 km DEM generated in this work using ICESat-2 surface height retrievals. The independent stake transect used to evaluate the RFs is shown in blue, and the 100-km snow radar transect shown in Figure 2 is shown in orange.


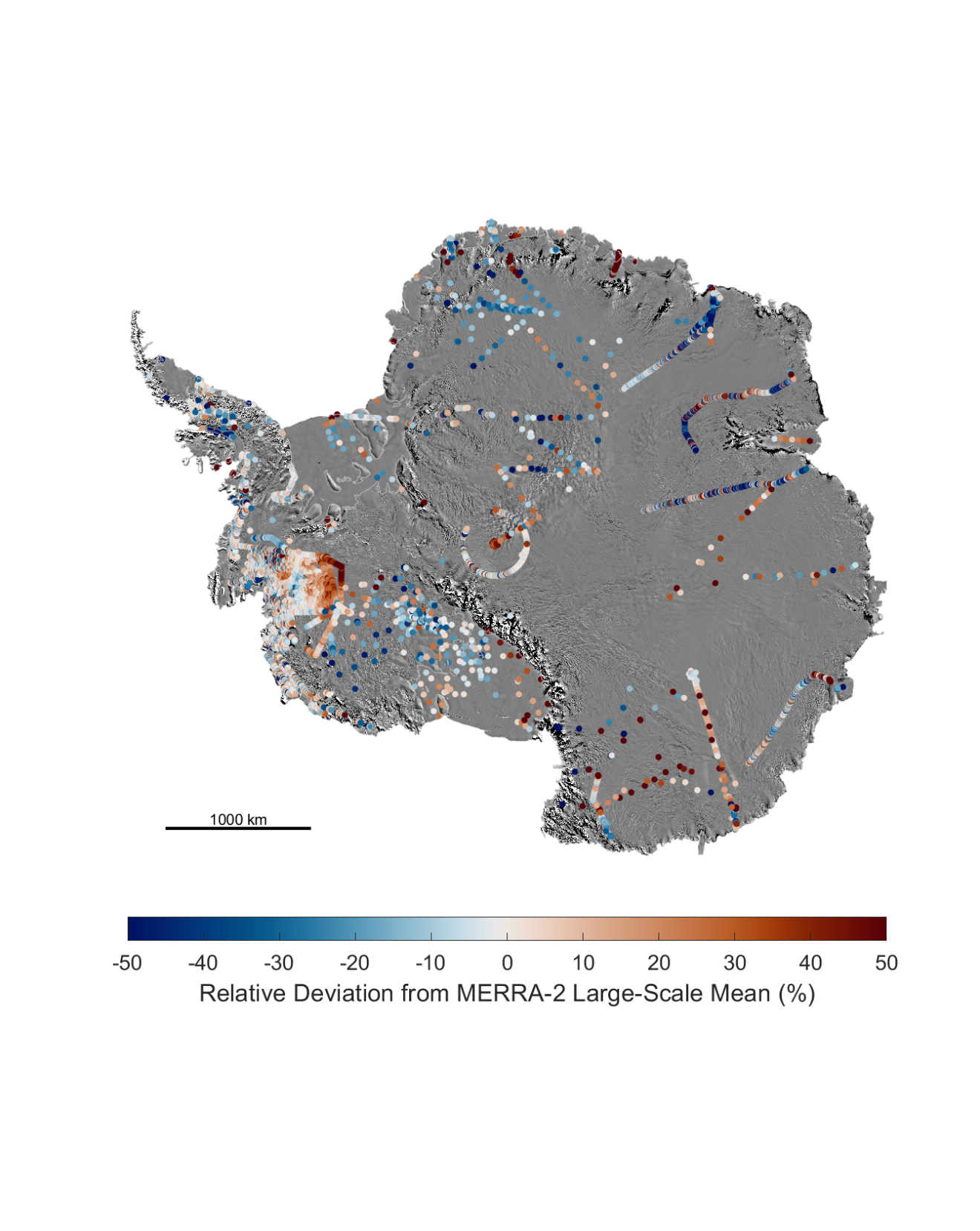
Figure S2. The relative deviation from the MERRA-2 large-scale net accumulation, or *SSV*, of each of the observations in both the GPR and traditional datasets. This plot is equivalent to the prediction plot in Figure 1a.


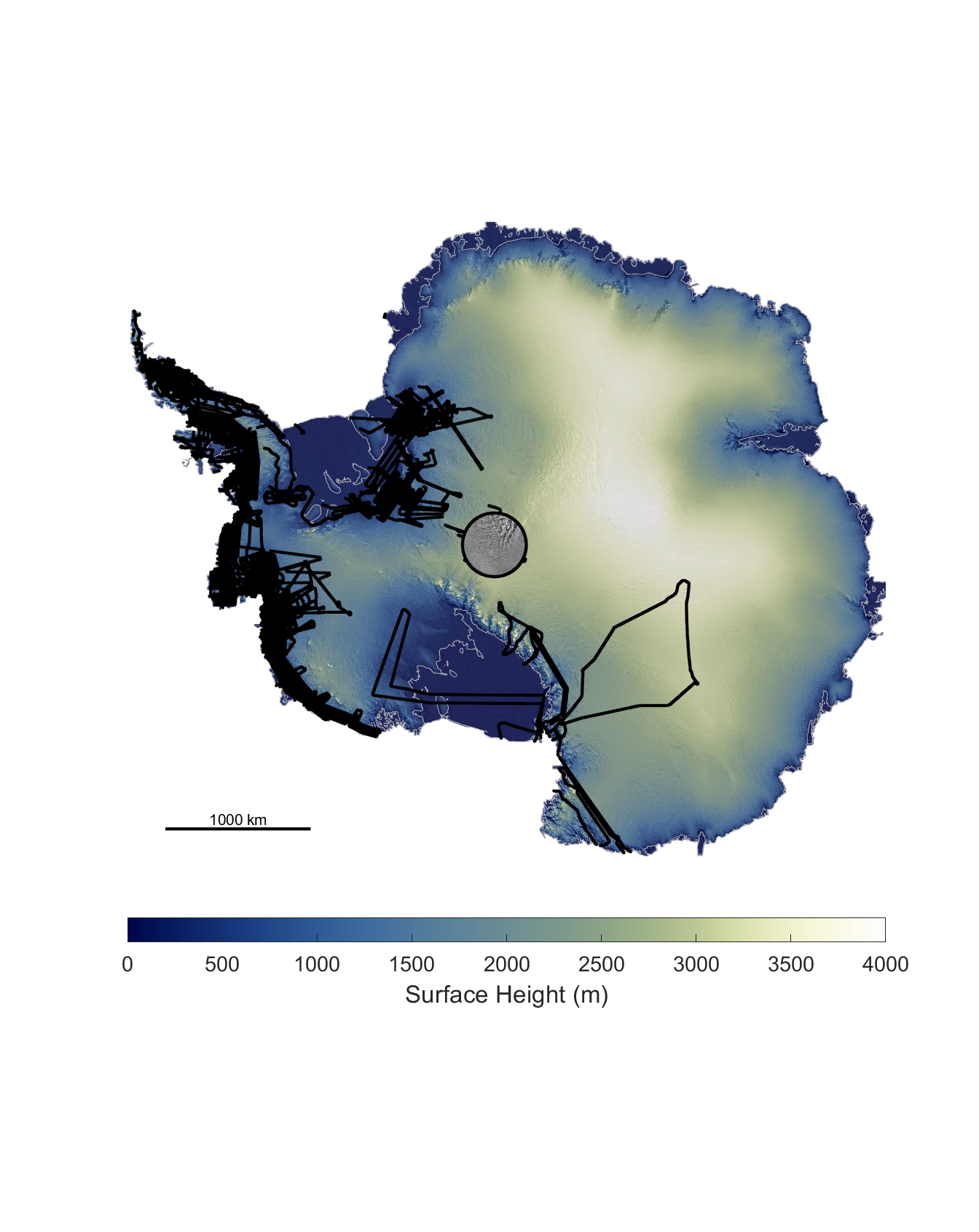


Figure S3. A 1 km DEM built using ICESat-2 ATL06 land ice heights in meters using the first 3 orbital cycles with OIB ATM data overlaid in black.


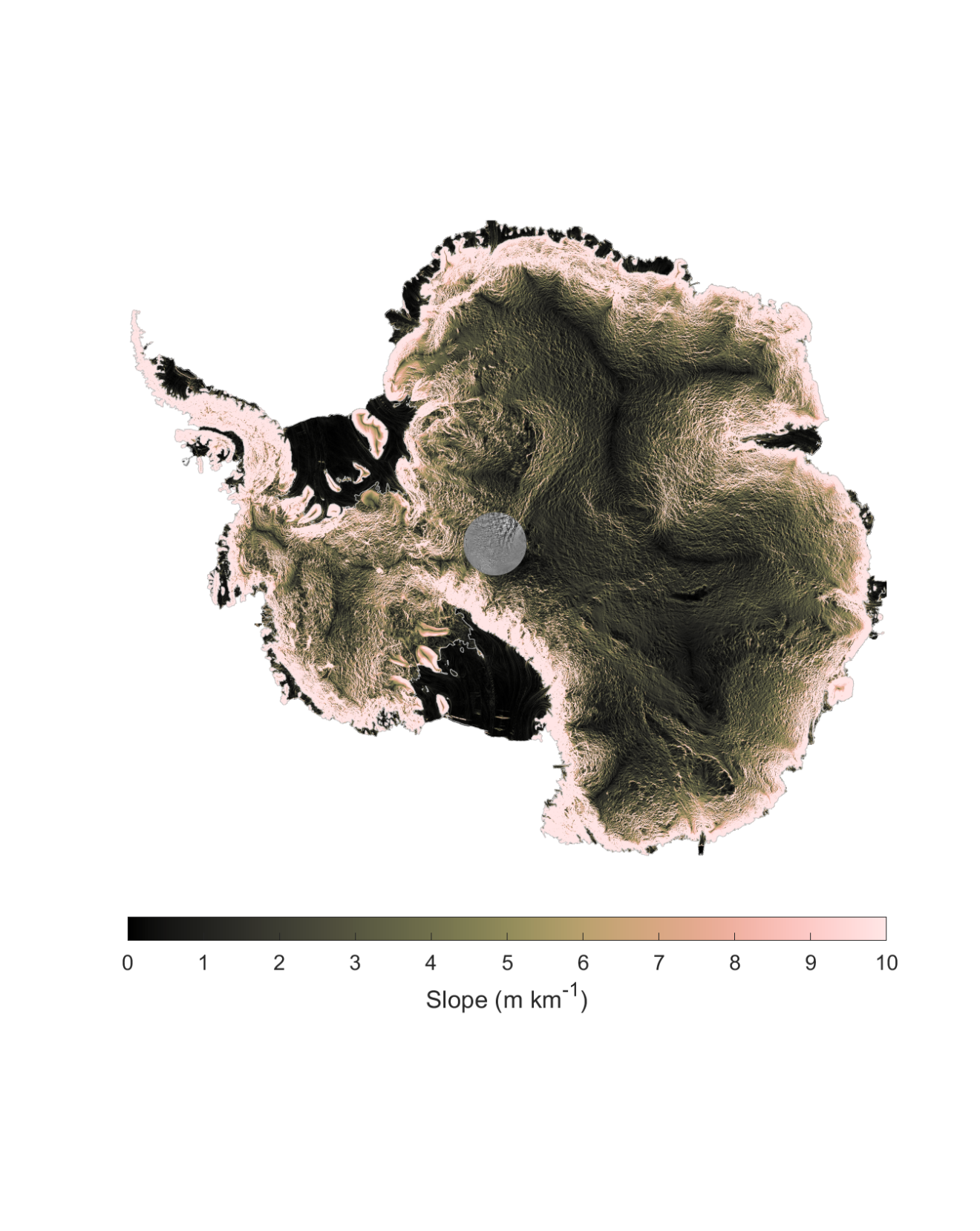


Figure S4. Surface slope derived from the ICESat-2 DEM in Figure S1.


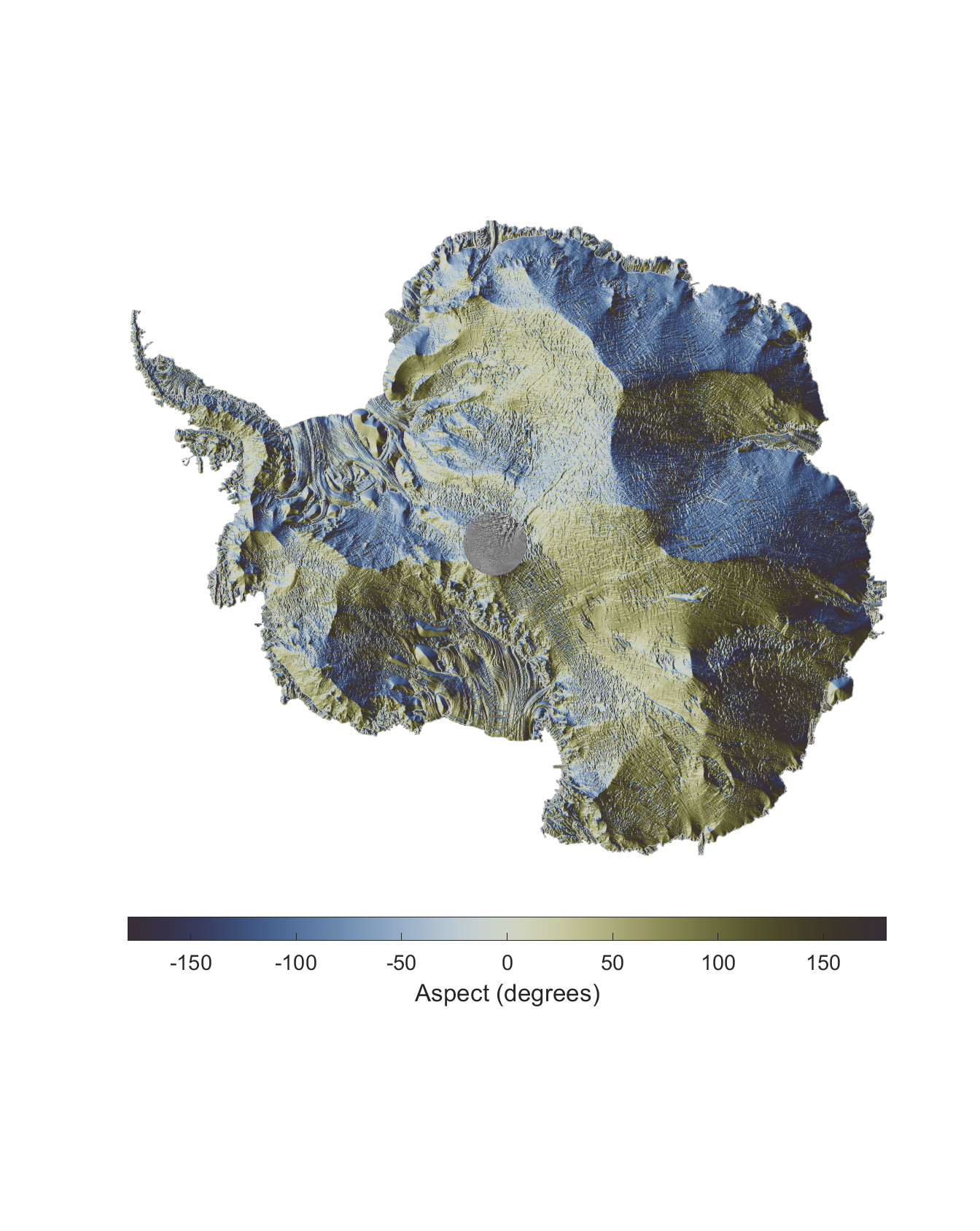


Figure S5. Aspect derived from the ICESat-2 DEM in Figure S1.


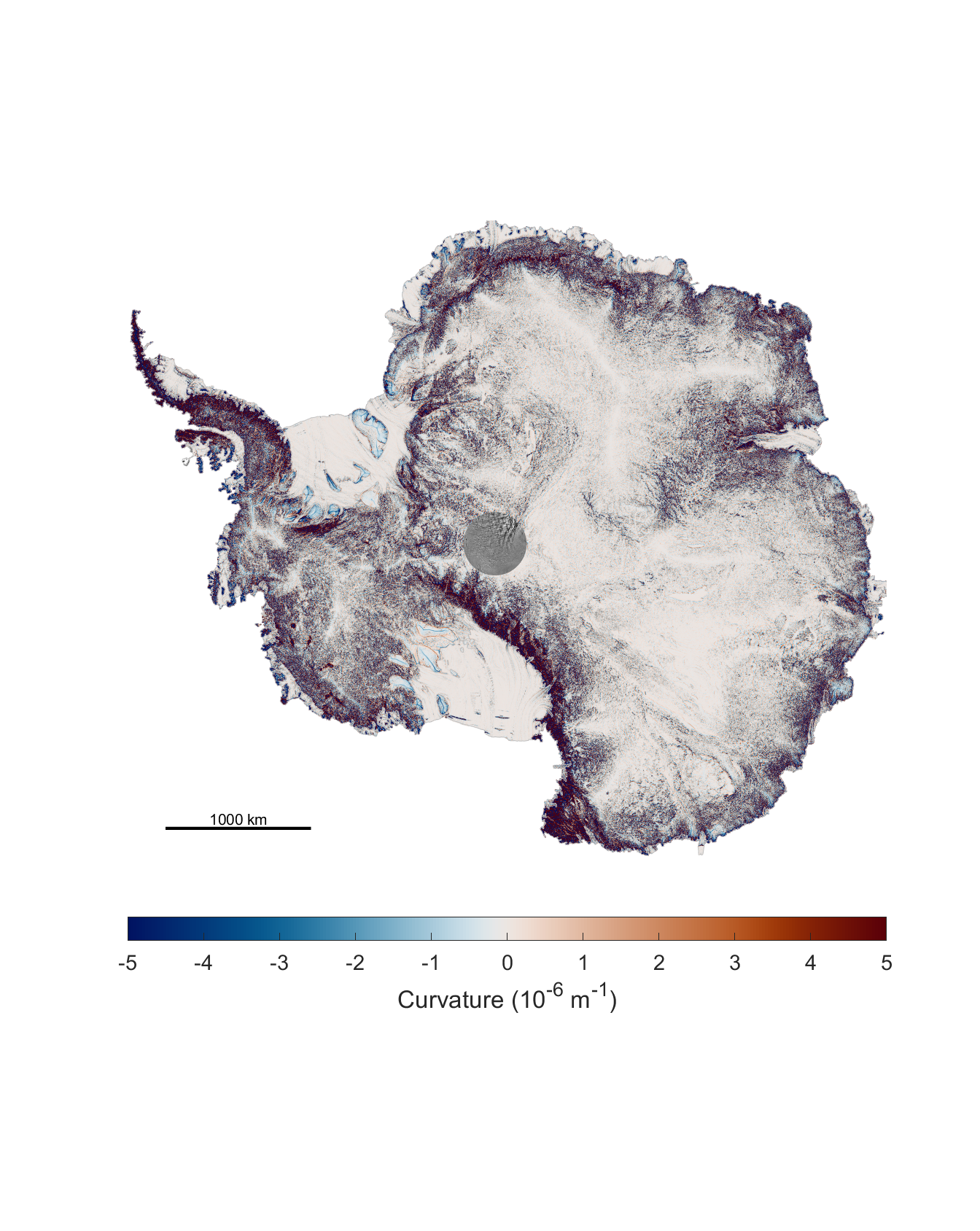


Figure S6. Curvature derived from the ICESat-2 DEM in Figure S1.


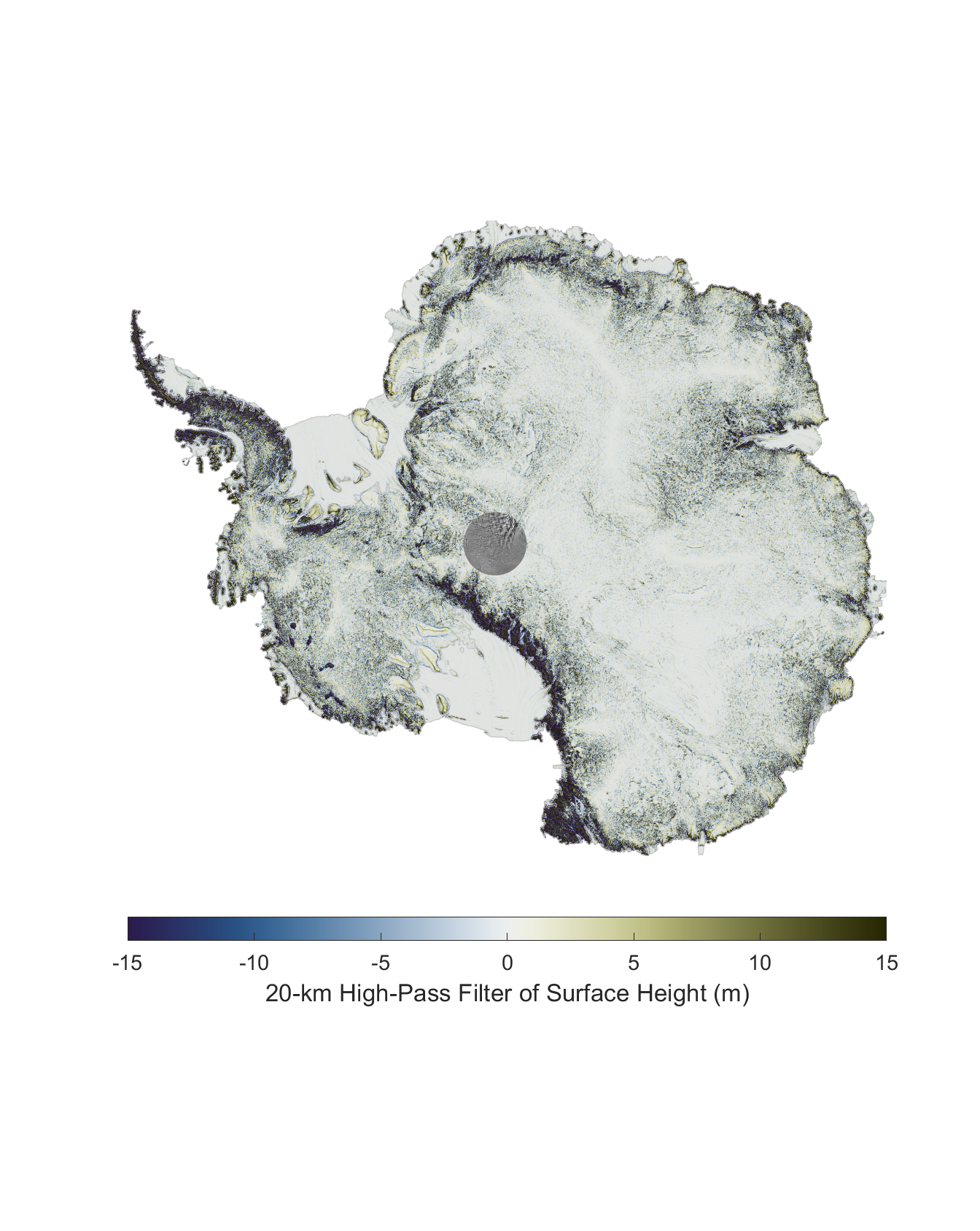


Figure S7. The ICESat-2 DEM in Figure S1 with a 20 km high-pass filter applied.


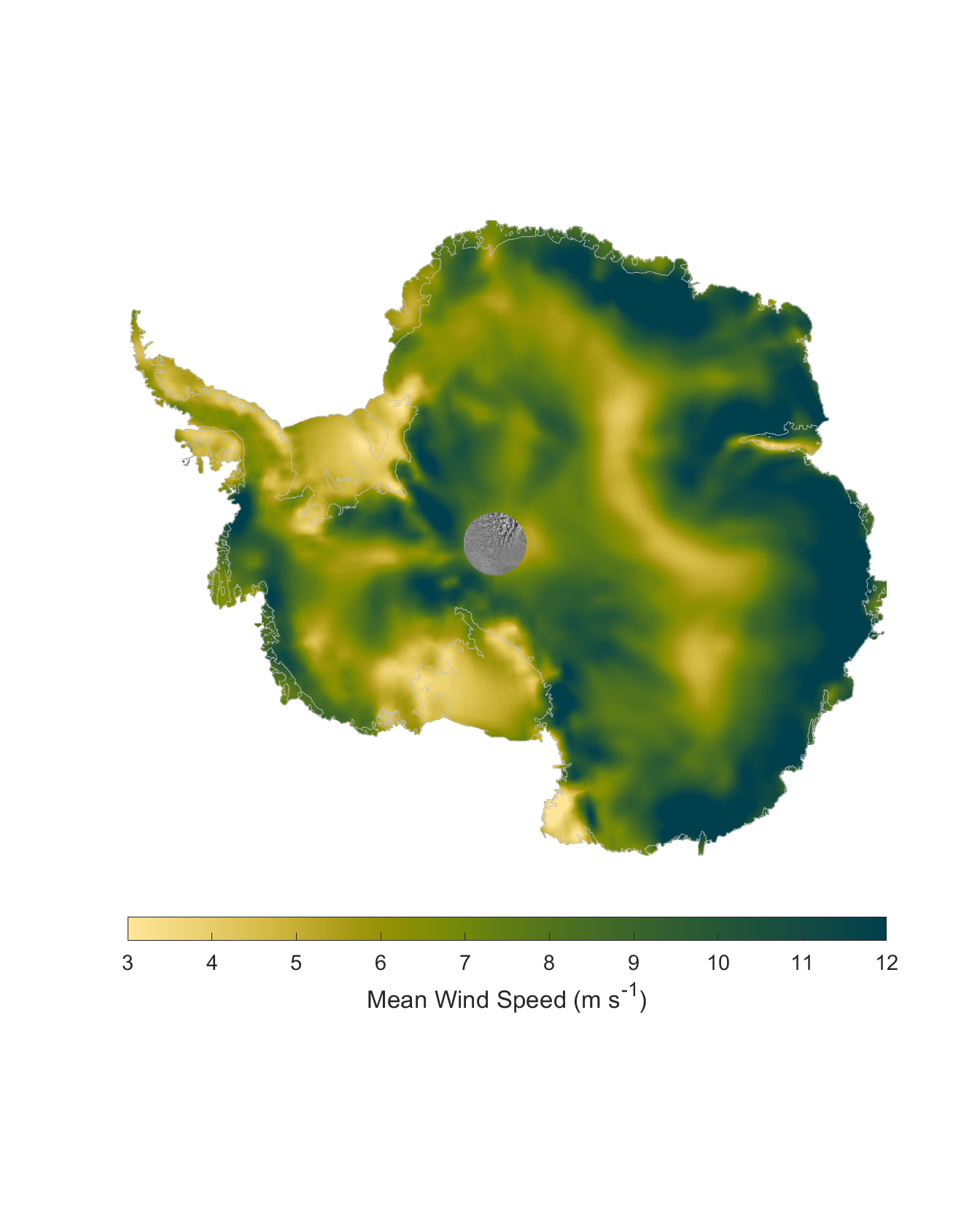


Figure S8. MERRA-2 mean annual 10-meter wind speed.


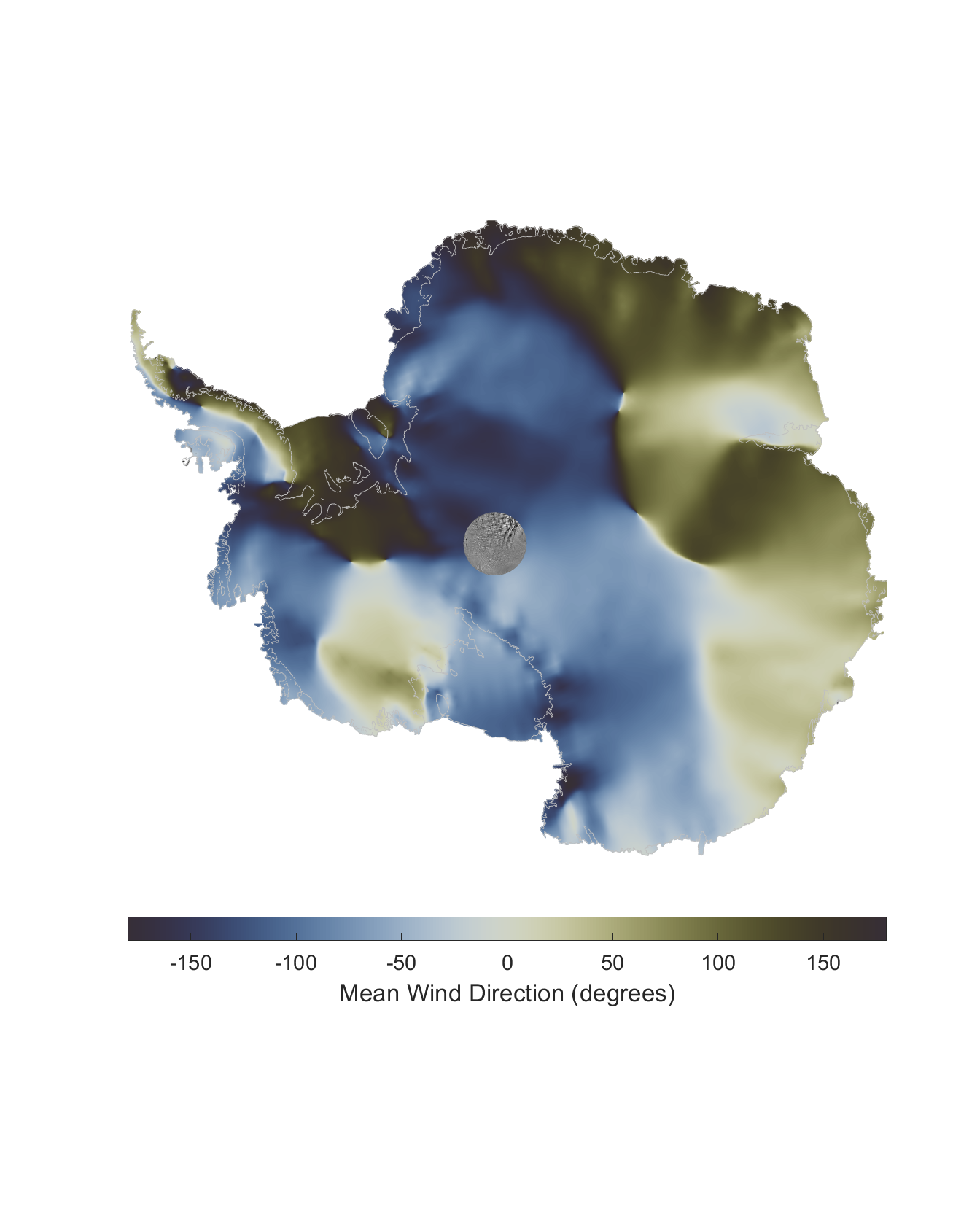


Figure S9. MERRA-2 mean annual 10-meter wind direction.


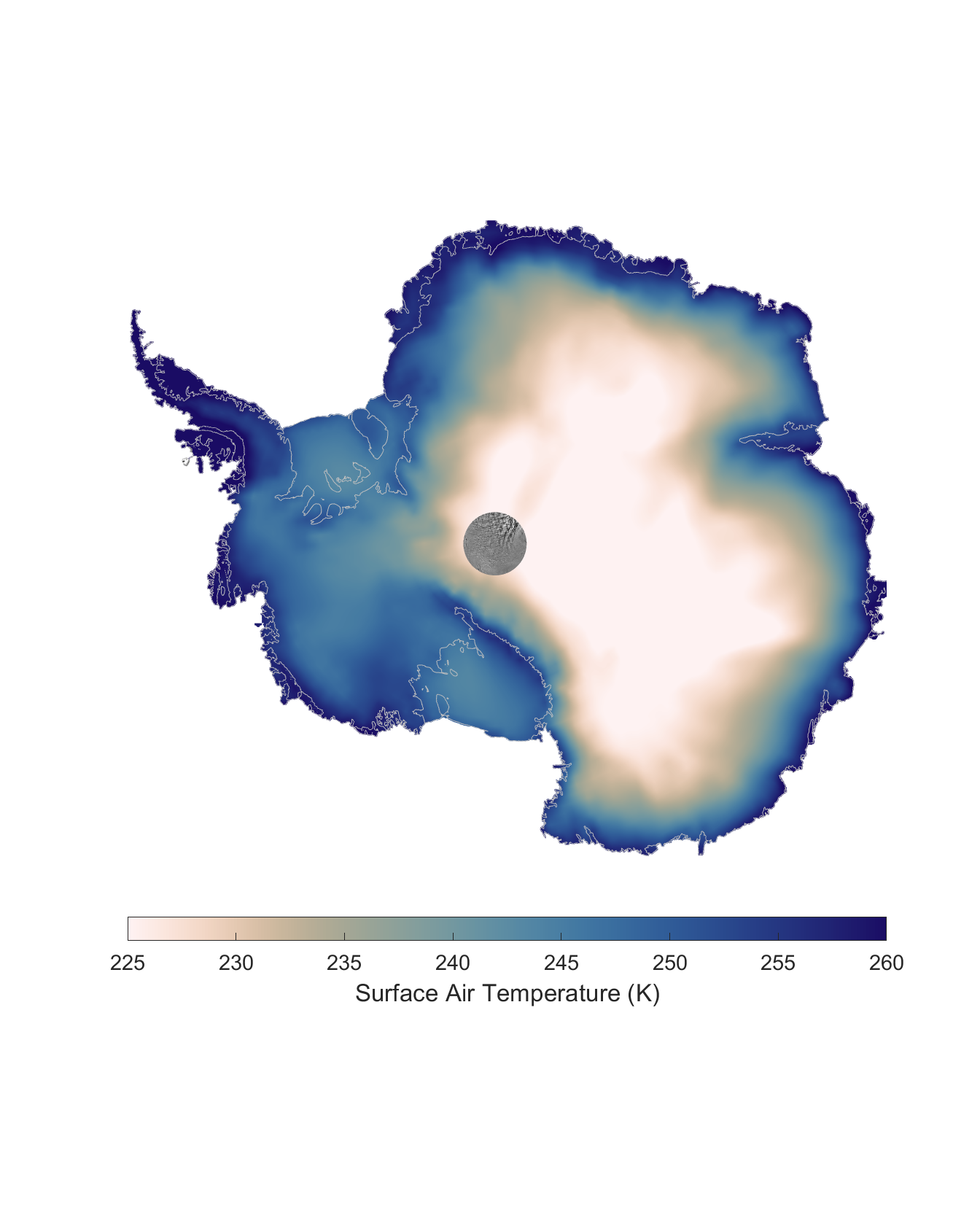


Figure S10. MERRA-2 mean annual surface air temperature.


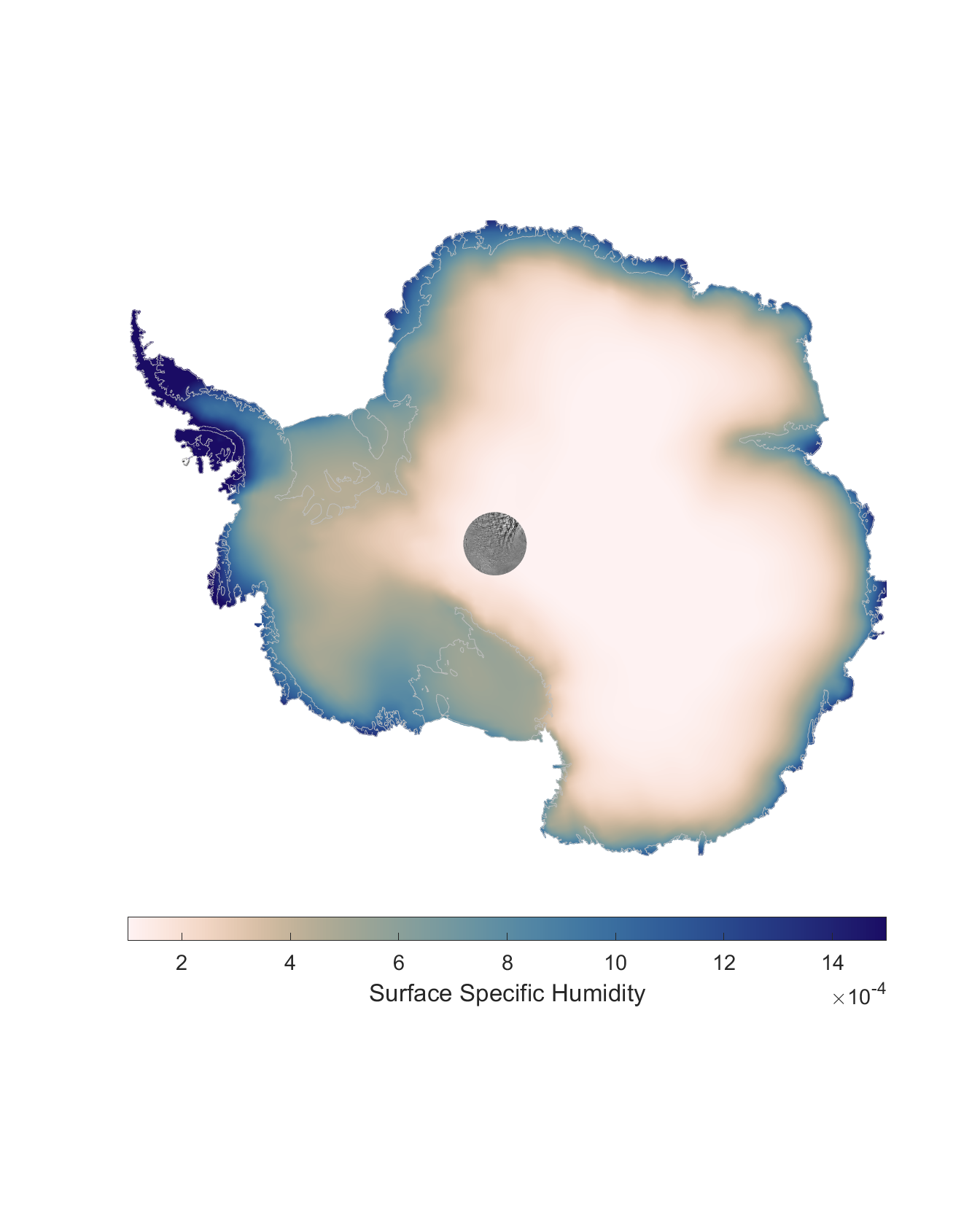


Figure S11. MERRA-2 mean annual surface specific humidity (kg kg^-1^).


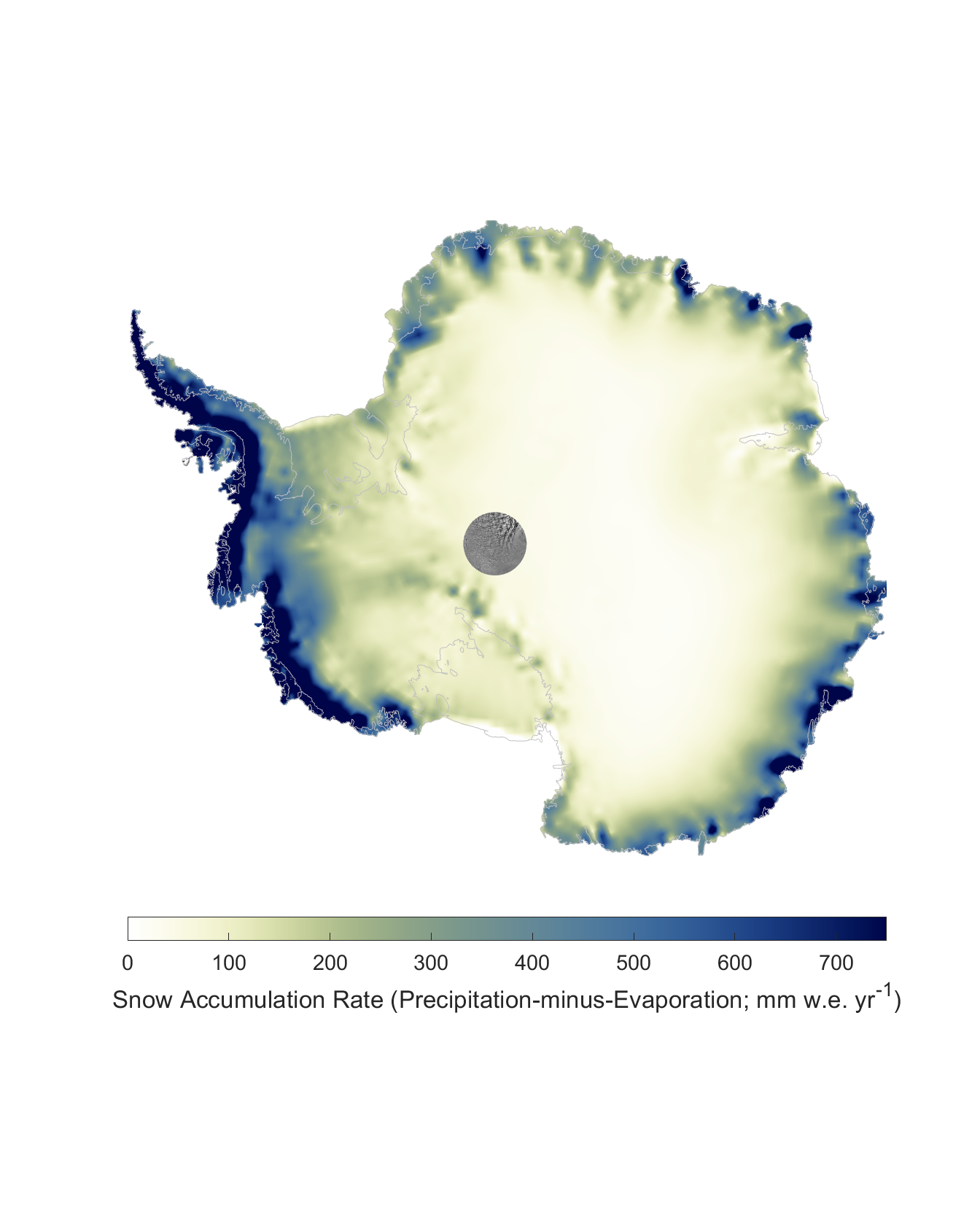


Figure S12. MERRA-2 mean annual precipitation-minus-evaporation (net accumulation).


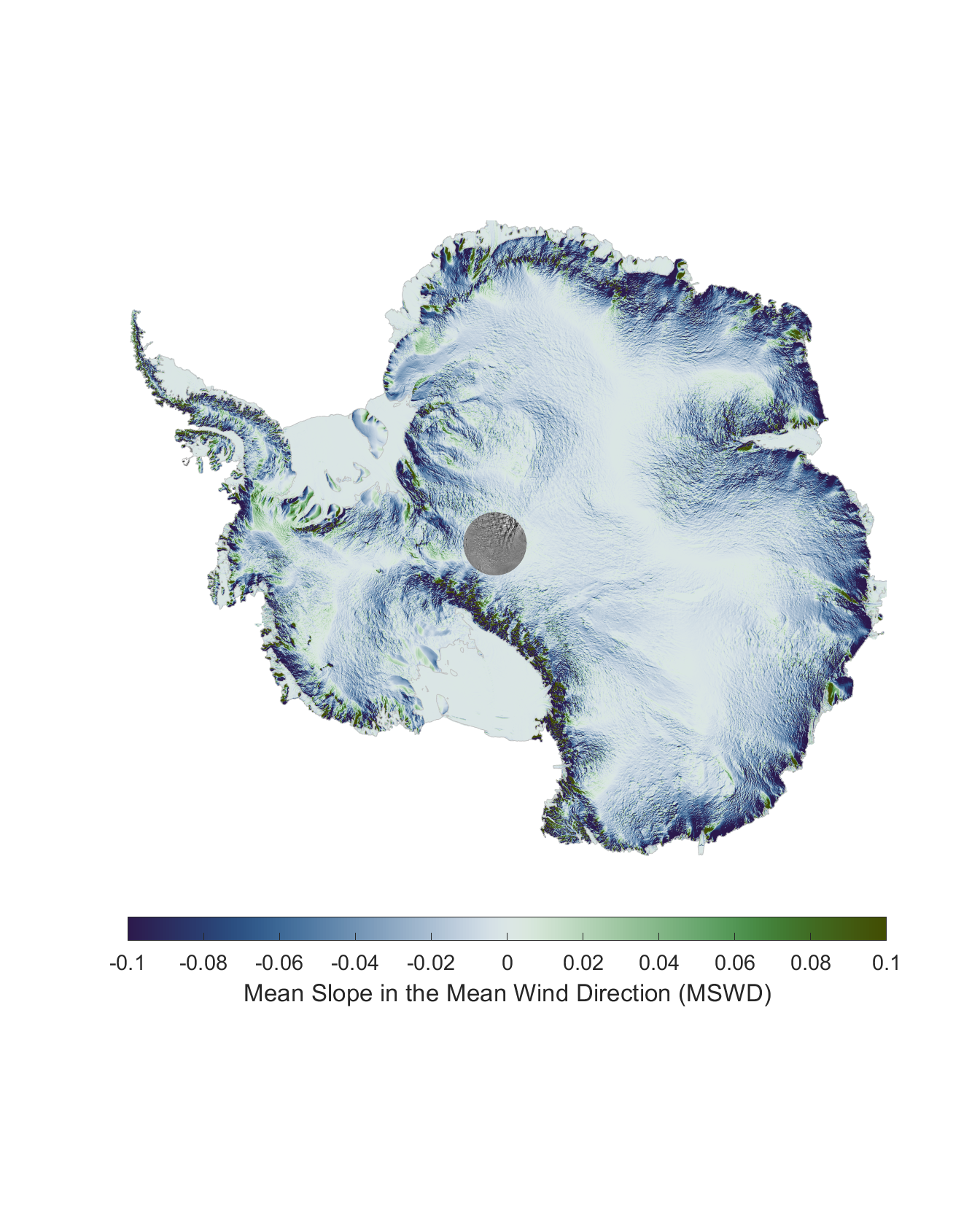


Figure S13. The modified mean slope in the mean wind direction (MSWD), the dot product of the wind and slope vectors, as described in the Section S3.


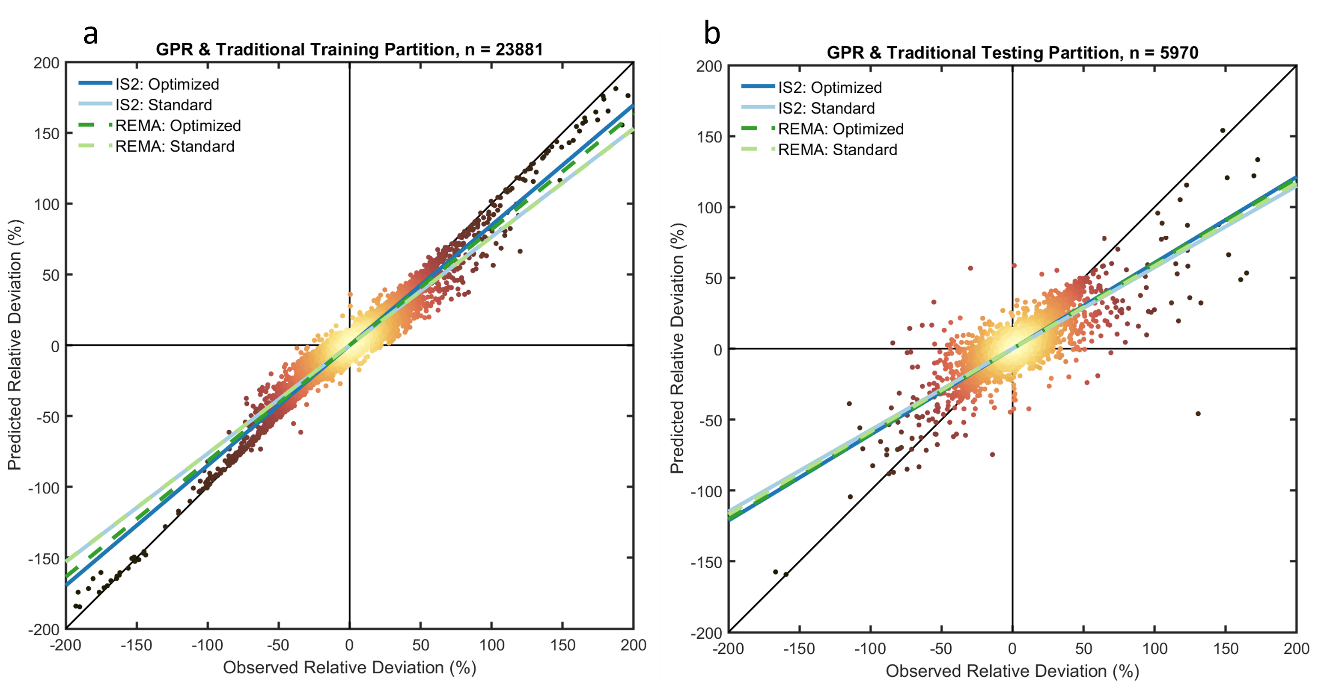


Figure S14. Comparison of GPR and traditional observations and RF predicted snow accumulation deviations from the large-scale mean for the (a) training and (b) testing partitions. Shading of points reflects density where brighter colors are representative of more data points. See Section S4.2.


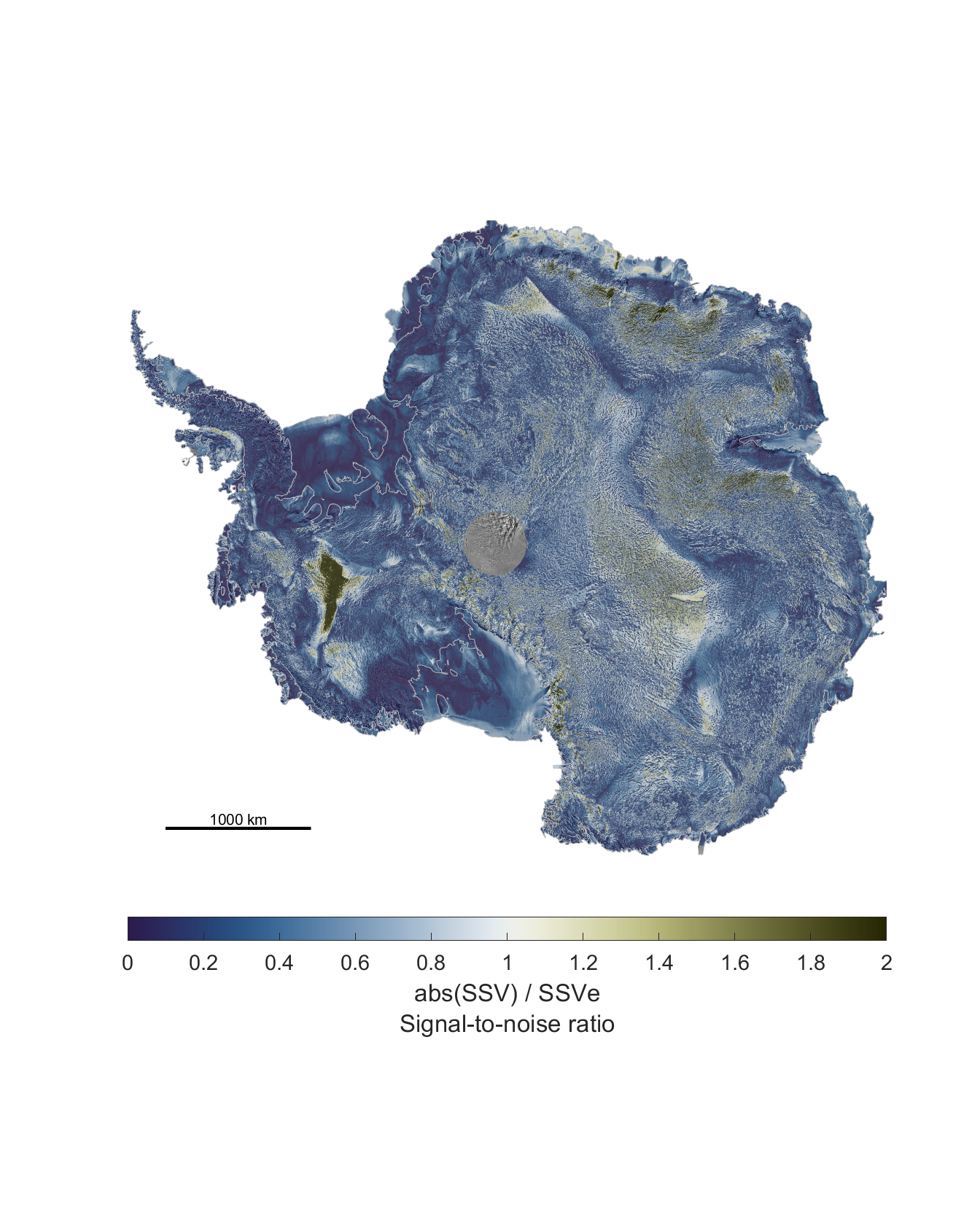


Figure S15. The signal-to-noise ratio of our combined predictions of SSV based on the predicted errors (SSVe). Only 11% of the ice sheet has a signal-to-noise ratio greater than one.


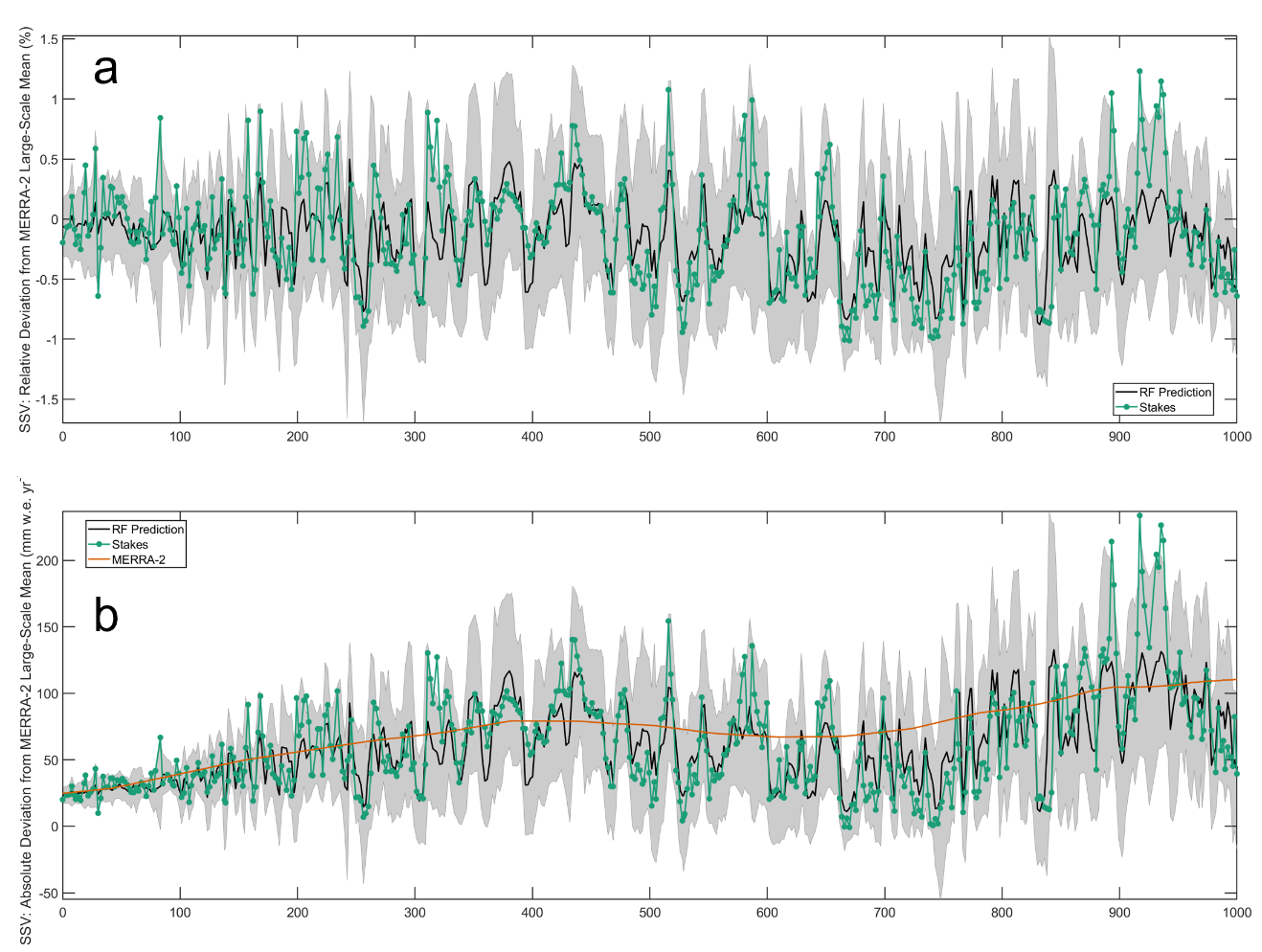


Figure S16. (a) Comparison of the RF *SSV* prediction in black, with the uncertainty bounds shaded in grey, and the independent stake transect in green (blue line in Figure S1b). (b) The same as (a) but presented as absolute accumulation rates from the RF model, the stake transect, and the large-scale MERRA-2 accumulation (orange line). Because (a) is in reference to the MERRA-2 large-scale accumulation, we do not plot the MERRA-2 values as they would simply all be zero.

|  | | | | | | | | | | |  |
| --- | --- | --- | --- | --- | --- | --- | --- | --- | --- | --- | --- |
|  | 2009-2018, *n* = 162,680 | | | | | 2018, *n* = 16,181 | | | | |  |
|  | *Lower Decile (10%)* | *Lower Quartile (25%)* | *Median* | *Upper Quartile (75%)* | *Upper Decile (90%)* | *Lower Decile (10%)* | *Lower Quartile (25%)* | *Median* | *Upper Quartile (75%)* | *Upper Decile (90%)* |  |
| ICESat-2 (this study)^3^ | -11.68 | -3.63 | -0.31 | 1.30 | 5.21 | -3.98 | -1.27 | -0.05 | 1.13 | 3.64 |  |
| CryoSat-2 (Helm)^1^ | -4.28 | -0.75 | 2.28 | 12.16 | 40.12 | -1.85 | 0.10 | 3.81 | 12.30 | 30.71 |  |
| CryoSat-2 (Slater)^1^ | -7.87 | -2.21 | 0.27 | 6.97 | 56.49 | -3.71 | -0.42 | 2.06 | 7.46 | 18.44 |  |
| REMA (Howat)^2^ | -6.70 | -2.17 | -0.04 | 2.14 | 6.92 | -3.12 | -1.00 | 0.66 | 3.46 | 8.59 |  |
| ^1^Helm et al. (2014) and Slater et al. (2018) were derived using ESA’s CryoSat-2 radar altimeter data | | | | | | | | | | |  |
| ^2^REMA was generated using commercial-satellite high-resolution stereophotogrammetry (Howat et al., 2019) | | | | | | | | | | |  |
| ^3^This work used NASA's ICESat-2 laser altimeter data; gaps were filled by REMA, but not used for this comparison | | | | | | | | | | |  |

Table S1. Evaluation of DEMs against Operation IceBridge Airborne Topographic Mapper elevation retrievals using all data (2009-2018) and the most recent year only (2018).

|  | **ICESat-2 DEM** | | | | | | **REMA** | | | | | |
| --- | --- | --- | --- | --- | --- | --- | --- | --- | --- | --- | --- | --- |
|  | **Optimized** | | | **Standardized** | | | **Optimized** | | | **Standardized** | | |
| min. leaf size | 1 | | | 5 | | | 2 | | | 5 | | |
| max. number of splits | 4600 | | | 4600 | | | 4200 | | | 4200 | | |
| # of predictors to sample | 6 | | | 3 | | | 4 | | | 3 | | |
| **Out of Bag Error (%)** |  |  |  |  |  |  |  |  |  |  |  |  |
| GPR | 0.98 | | | 1.04 | | | 1.01 | | | 1.04 | | |
| Traditional | 7.67 | | | 8.04 | | | 7.27 | | | 7.43 | | |
| GPR + Traditional | 2.43 | | | 2.45 | | | 2.48 | | | 2.48 | | |
| ***Integrated Mass Flux (Gt yr^-1^)*** |  |  |  |  |  |  |  |  |  |  |  |  |
| Grounded | -19.8 ± 8.0 | | | -35.4 ± 7.8 | | | -18.8 ± 8.4 | | | -19.1 ± 7.9 | | |
| Floating | 14.9 ± 5.9 | | | 12.1 ± 5.6 | | | 21.6 ± 6.0 | | | 22.4 ± 5.8 | | |
| Total | -4.9 ± 9.9 | | | -23.3 ± 9.6 | | | 2.8 ± 10.3 | | | 3.3 ± 9.8 | | |
| ***R^2^*** | Train | Test | Transect | Train | Test | Transect | Train | Test | Transect | Train | Test | Transect |
| GPR | 0.90 | 0.67 |  | 0.86 | 0.65 |  | 0.88 | 0.67 |  | 0.86 | 0.65 |  |
| Traditional | 0.99 | 0.67 | 0.44 | 0.94 | 0.66 | 0.45 | 0.98 | 0.67 | 0.41 | 0.94 | 0.67 | 0.42 |
| GPR + Traditional | 0.94 | 0.67 |  | 0.89 | 0.66 |  | 0.92 | 0.67 |  | 0.89 | 0.66 |  |
| ***RMSE (%)*** |  |  |  |  |  |  |  |  |  |  |  |  |
| GPR | 4.38 | 7.21 |  | 5.01 | 7.15 |  | 4.66 | 7.11 |  | 4.99 | 7.11 |  |
| Traditional | 4.47 | 19.66 | 23.50 | 9.37 | 18.61 | 21.70 | 5.80 | 19.51 | 23.30 | 9.46 | 18.96 | 23.00 |
| GPR + Traditional | 4.72 | 8.97 |  | 5.60 | 8.74 |  | 5.03 | 8.86 |  | 5.60 | 8.76 |  |
| ***Slope*** |  |  |  |  |  |  |  |  |  |  |  |  |
| GPR | 0.78 | 0.62 |  | 0.73 | 0.59 |  | 0.76 | 0.60 |  | 0.73 | 0.59 |  |
| Traditional | 0.95 | 0.59 | 0.53 | 0.82 | 0.55 | 0.50 | 0.91 | 0.60 | 0.50 | 0.82 | 0.58 | 0.50 |
| GPR + Traditional | 0.85 | 0.61 |  | 0.76 | 0.57 |  | 0.82 | 0.60 |  | 0.76 | 0.58 |  |

Table S2. Characteristics of each random forest regression model of small-scale variability in accumulation and out-of-box and independent testing evaluation statistics. The minimum leaf size, maximum number of splits, and number of predictors to sample provide the RF parameters used for each of the four models, which are separated by the DEM used (ICESat-2 or REMA) and whether the parameters were optimized or if they are standard practice. We break the performance statistics into three categories: GPR only, traditional only, and then their combination to allow assessment of performance across measurement types. The out-of-bag errors are generated by evaluation of training data that are left out of each tree when conducting sampling with replacement (for more details see Section S4.2) and are likely underestimates of performance, especially in non-surveyed regions. The integrated impact of the SSV map on net accumulation is also provided, along with its uncertainty which accounts for spatial correlations up to 20 km and is broken down between floating and grounded ice. The R2, RMSE, and slope are calculated through linear regression between the training\testing\transect observations and the predicted SSV (see Figure S14).
